# Severe, but not moderate asthmatics share blood transcriptomic changes with post-traumatic stress disorder and depression

**DOI:** 10.1371/journal.pone.0275864

**Published:** 2022-10-07

**Authors:** Sandor Haas-Neill, Anna Dvorkin-Gheva, Paul Forsythe

**Affiliations:** 1 The Brain Body Institute, St. Joseph’s Hospital, McMaster University, Hamilton, Ontario, Canada; 2 McMaster Immunology Research Centre, Department of Medicine, McMaster University, Hamilton, Ontario, Canada; 3 Alberta Respiratory Centre, Division of Pulmonary Medicine, Department of Medicine, University of Alberta, Edmonton, Alberta, Canada; University of Nebraska Medical Center, UNITED STATES

## Abstract

Asthma, an inflammatory disorder of the airways, is one of the most common chronic illnesses worldwide and is associated with significant morbidity. There is growing recognition of an association between asthma and mood disorders including post-traumatic stress disorder (PTSD) and major depressive disorder (MDD). Although there are several hypotheses regarding the relationship between asthma and mental health, there is little understanding of underlying mechanisms and causality. In the current study we utilized publicly available datasets of human blood mRNA collected from patients with severe and moderate asthma, MDD, and PTSD. We performed differential expression (DE) analysis and Gene Set Enrichment Analysis (GSEA) on diseased subjects against the healthy subjects from their respective datasets, compared the results between diseases, and validated DE genes and gene sets with 4 more independent datasets. Our analysis revealed that commonalities in blood transcriptomic changes were only found between the severe form of asthma and mood disorders. Gene expression commonly regulated in PTSD and severe asthma, included *ORMDL3* a gene known to be associated with asthma risk and STX8, which is involved in TrkA signaling. We also identified several pathways commonly regulated to both MDD and severe asthma. This study reveals gene and pathway regulation that potentially drives the comorbidity between severe asthma, PTSD, and MDD and may serve as foci for future research aimed at gaining a better understanding of both the relationship between asthma and PTSD, and the pathophysiology of the individual disorders.

## Introduction

Asthma is a chronic inflammatory disease of the airways associated with recurrent episodes of wheezing, shortness of breath, chest tightness, and coughing. Generally, asthma is characterized by reversible constriction of the airways in response to allergen, but it can also be triggered by viral infection, physical activity, stress, or a negative mood [1]. Asthma affects 300 million people worldwide and the World Health Organization has estimated that it is responsible for the loss of 15 million disability-adjusted life years (DALYs) annually [2, 3]. Asthma is also the most common chronic disease in children [4].

Epidemiological studies have shown significant association between asthma and mental health disorders, including anxiety, depression, panic attacks, and posttraumatic stress disorder (PTSD) [5–10].

MDD, more commonly referred to as ‘depression’ is a mental health disorder characterized by a low self-esteem, mood, and enjoyment of activities [11].

Studies have demonstrated consistent comorbidity between asthma and depression [7] and Youth with asthma are close to twice as likely to have anxiety and depressive disorders as those without asthma [8]. The co-occurrence of an anxiety or depressive disorder is associated with poor symptom control, impaired quality of life and increased health care utilization. While many studies have focused on psychosocial factors linking asthma and depression there is evidence that there may be shared pathophysiological factors between the diseases. For example, in a large-scale study in adults twins the association between depression and asthma remained significant after controlling for genetic and environmental factors [12]. However, the potential mechanisms and causality relating depression and asthma remain unclear [13–15].

PTSD is a mental health disorder that usually follows exposure to a traumatic event. The characteristic symptoms of PTSD include intrusive memories and nightmares, negative mood impaired cognition, avoidance behaviors, and changes to arousal behaviors such as increased irritability [16].

Clinical evidence supports a strong link between inflammatory conditions and PTSD with a particularly strong association between asthma and the prevalence and severity of PTSD [17, 18]. A twin study of Vietnam war veterans found that those with the top quartile of PTSD scores were 2-fold more likely to have asthma than those in the lower quartile [6]. This association was shown not to be predicted by familial or genetic factors, smoking, depression, or demographic factors [6]. Wisnivesky *et al*., (2021) [5] found that 19% of world trade center rescue and recovery workers with asthma also had PTSD, 10 times the prevalence in the general population. PTSD is also one of the greatest risk factors for decreased quality-of-life related to asthma [17, 18] and these poorer asthma outcomes do not appear to be due to differences in key asthma self-management behaviors [18]. Conversely, individuals with asthma prior to PTSD have been demonstrated to develop more aggravated asthma symptoms after the development of PTSD, while non-asthmatic subjects who develop PTSD have increased risk of adult onset asthma, suggesting a bidirectional relationship between these disorders [17].

An attempt by Jiang *et al*., (2014) [7] to identify a mechanism behind the comorbidity of asthma and MDD suggested immune factors may underlie both disorders. The investigation of 38 depression studies found that monocyte-derived, and other inflammatory cytokines (IL-1, IL-4, IL-6, and TNF) were significantly overexpressed in individuals with depression, while T cell derived cytokines (IL-10, and INF-γ) were uncorrelated with depression. Data comparing CD4+ T-cell expression in asthmatics with and without depression has also shown that 156 of 1448 total identified genes were differentially expressed in the depressed asthmatics group [19], suggesting that in circulating T-cells there is a unique transcriptomic profile for comorbid asthma and depression.

Genome-wide association studies (GWAS) have identified some shared genetic traits between those with asthma and MDD [20, 21]. In a cross-trait meta-analysis, Zhu *et al*., (2019) [20] identified 10 genomic loci shared between asthma and MDD and mendelian randomization identified a significant causal effect of MDD on asthma. The cross-trait meta-analysis performed by Cao *et al*., (2021) [21] identified 18 loci jointly associated between MDD and atopic diseases (asthma, eczema, and hay fever). Through Mendelian randomization analysis the investigators found that MDD confers a stronger causal effect on those atopic diseases than they confer on MDD.

Similarly, in a meta-analysis by Nievergelt *et al*., (2019) [22], a pairwise genetic correlation demonstrated a high association between PTSD and asthma. Chronic stress, maternal stress, and more fundamentally, oxidative stress are also associated with severe asthma and increased asthma exacerbations [23–26]. Yan *et al*., 2021 [24] identified 12 genes methylated in individuals with exposure to chronic stress and violence, that were then shown to be associated with atopic childhood asthma. Although these studies were not looking at PTSD specifically, it is likely that genes associated with violence and chronic stress exposure would have close ties to those associated with PTSD.

Here, we downloaded 5 publicly available datasets from GEO, each of which compare one of PTSD, MDD, or asthma (a very large dataset which we split randomly into 2 datasets) blood transcription to that of healthy subjects. One dataset of each disease was used to explore genes and gene sets commonly shared between diseased subjects, and the other of each disease dataset was used to validate the genes and sets identified. Prior to conducting the investigation, we were interested in transcription specifically, as it facilitates functional change in the body and therefore we decided to compare the data to the hallmark, and C2 gene sets, which characterize canonical and curated changes in the body. Additionally, we hypothesized that as Jiang *et al*., (2014) [7] found immune factors involved in comorbidity, immune transcriptional changes commonly differentiated in whole blood would delineate the source of comorbidity. Immune factors have also been found partially responsible for cross talk between gut and brain in psycho-active probiotic treated mice exhibiting mood disorder-like symptoms [27–29]. For these reasons, we also compared these datasets to the C7 - immune signature gene set.

With a deeper understanding of the established comorbidity between mental health disorders and asthma, may come tangible knowledge on how to combat the root cause of these diseases and an expectation for how treatment of one disorder might affect another. Therefore, the goal of this study was to expand on genome-wide association studies by using publicly available data to characterize transcriptomic similarities between these disorders through analysis of genes and gene sets commonly differentially expressed between those suffering from the diseases and healthy subjects.

## Results

### Exploration of commonly differentially expressed genes

**Fig 1:**
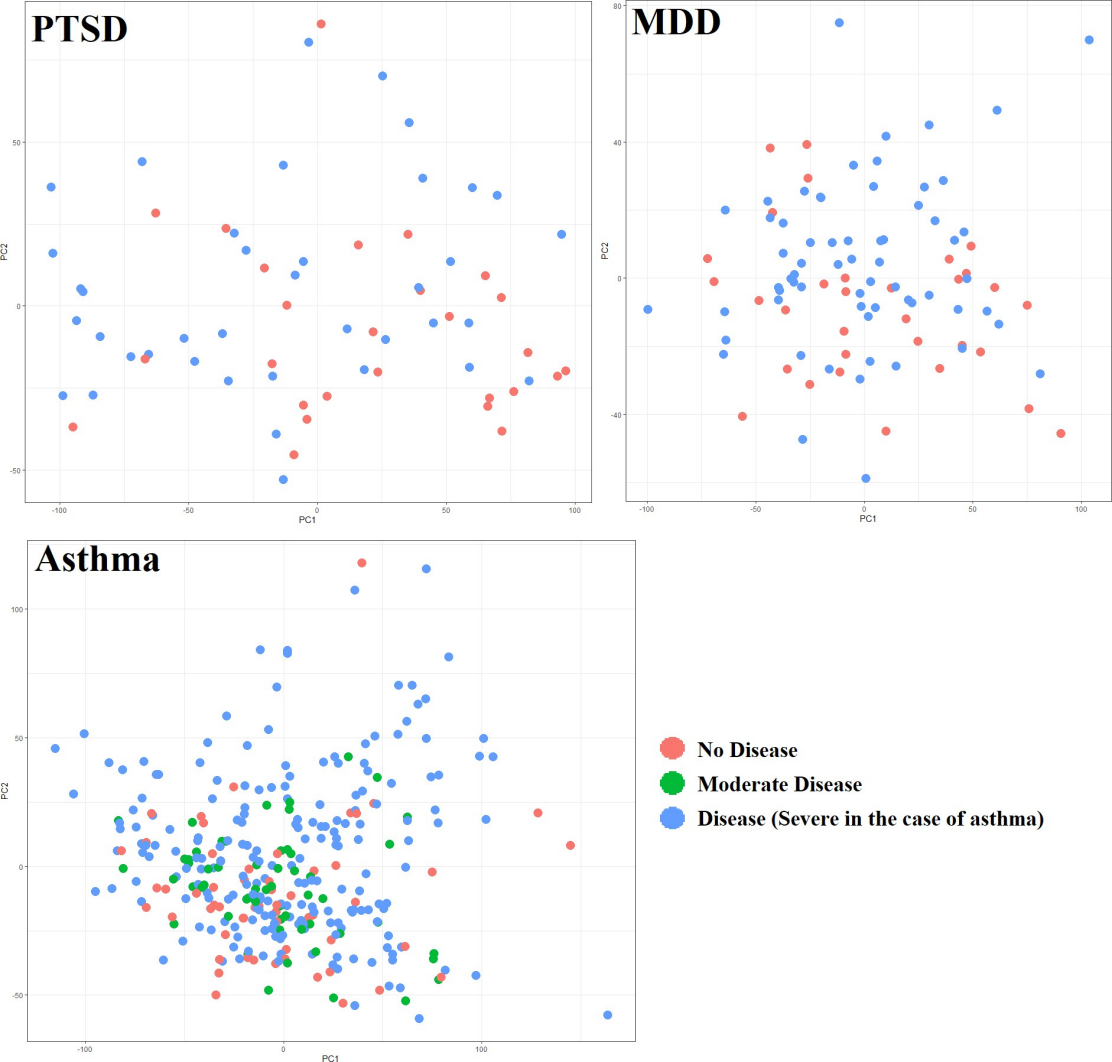
Principal component analysis. (PCA) showing PC1 and PC2 in each of the 3 disease exploration datasets.

The 3 exploration datasets first underwent hierarchical clustering analysis, but there were no distinct clusters formed pertaining to diseased vs healthy subjects or along the lines of any other collected meta data. Principal component analysis was then used to check that no known variables could account for major differences that may arise during DE and GSEA analysis (Fig 1). There was no apparent grouping along PC1 or PC2 for any of the datasets, including for diseased vs healthy subjects (Fig 1). For the PTSD exploration cohort, 40.7% of the variance was accounted for by PC1, and 10.6% by PC2; for MDD, 16.8% of the variance was accounted for by PC1, and 6.2% by PC2; and for asthma, 22.7% of the variance was explained by PC1, and 10.0% by PC2.

**Fig 2:**
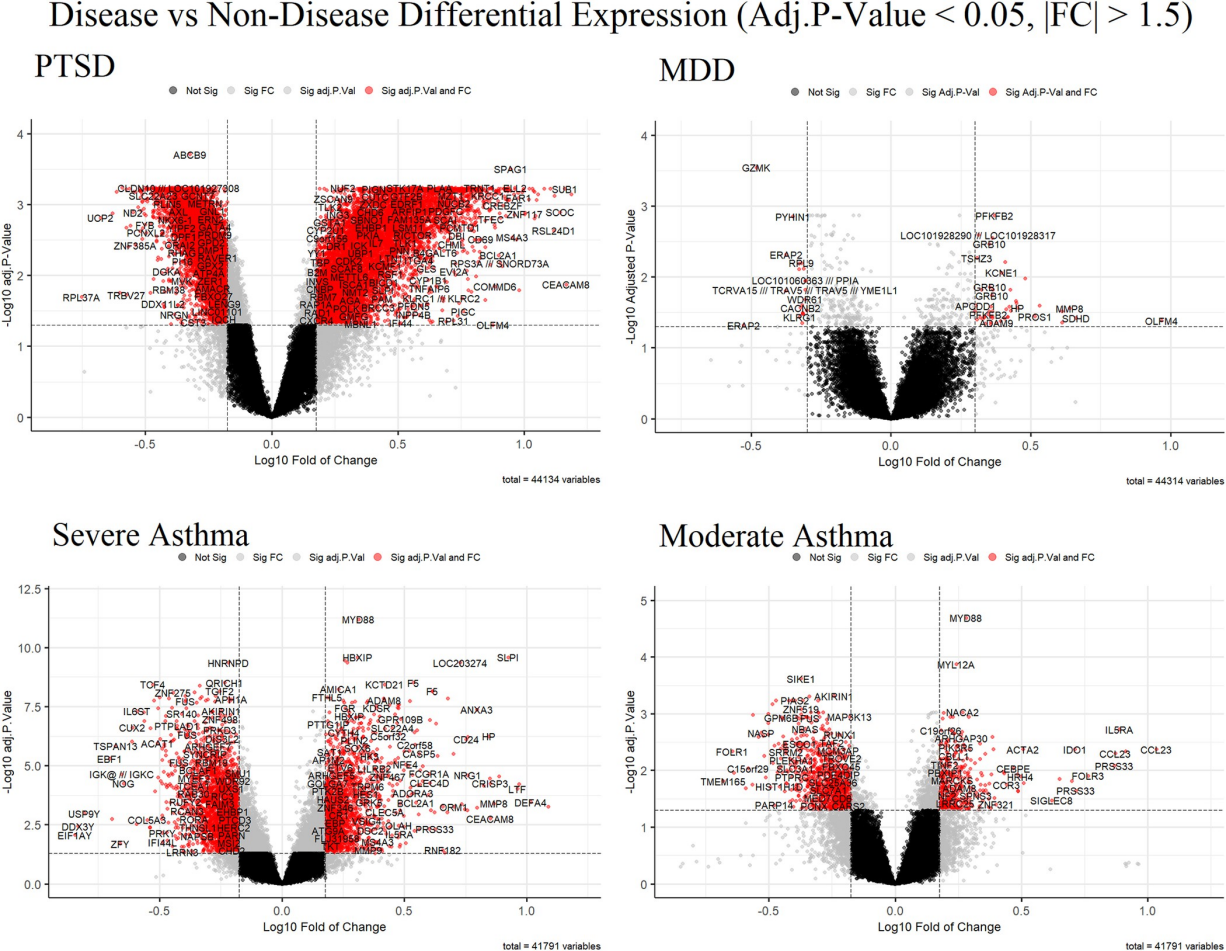
mRNA from the blood of subjects with a disease (PTSD, MDD, severe asthma, and moderate asthma) were compared to blood mRNA from non-diseased subjects for each exploration cohort dataset. The vertical threshold denotes genes or transcripts that are statistically significant (adjusted p-value < 0.05) while the horizontal threshold denotes genes or transcripts with an absolute fold change greater than 1.5. Genes or transcripts that meet none of these criteria are black, one of these criteria are grey, and both are red. The red genes, found to be significant, are also shown next to their symbols.

Differential expression analysis of each disease to control subjects from their respective datasets reveals significant differences in both genes being up- and downregulated in all diseases (Fig 2). The analysis identified 8,321, 208, 1,736, and 373 genes significantly upregulated (adjusted p-value < 0.05; FC ≥ 1.5) in PTSD, MDD, severe asthma, and moderate asthma respectively, as compared to the corresponding controls. 7,062, 294, 2,735, and 901 genes were found to be significantly downregulated (adjusted p-value < 0.05; FC ≤ -1.5) in the same comparisons respectively.

**Fig 3:**
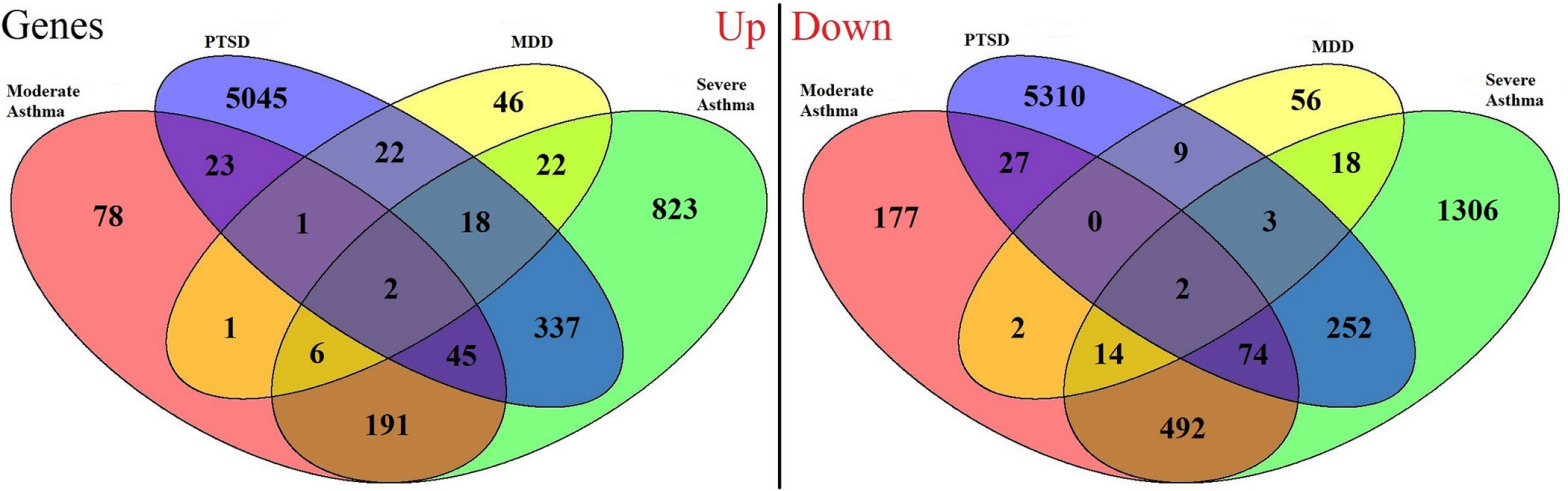
The number of genes differentially expressed from healthy subjects in the same direction between different diseases. The numbers within the different overlaps of the venn diagram are the number of genes significantly (adjusted P-value < 0.05) differentially expressed in both exploration datasets. For example, in the left ‘up’ panel, there are 22 genes in the PTSD and MDD exploration sets that are similarly significantly overexpressed, and in the right ‘down’ panel, there are 2 genes commonly underexpressed in all disease exploration datasets compared to their respective healthy controls.

Significantly regulated (adjusted P-value < 0.05, |FC| ≥1.5) genes were compared between the exploration datasets for each disease. Genes found commonly to be regulated in the same direction in patients relatively to the healthy controls for multiple diseases were plotted in (Fig 3).

### Exploration of commonly regulated gene sets

**Fig 4:**
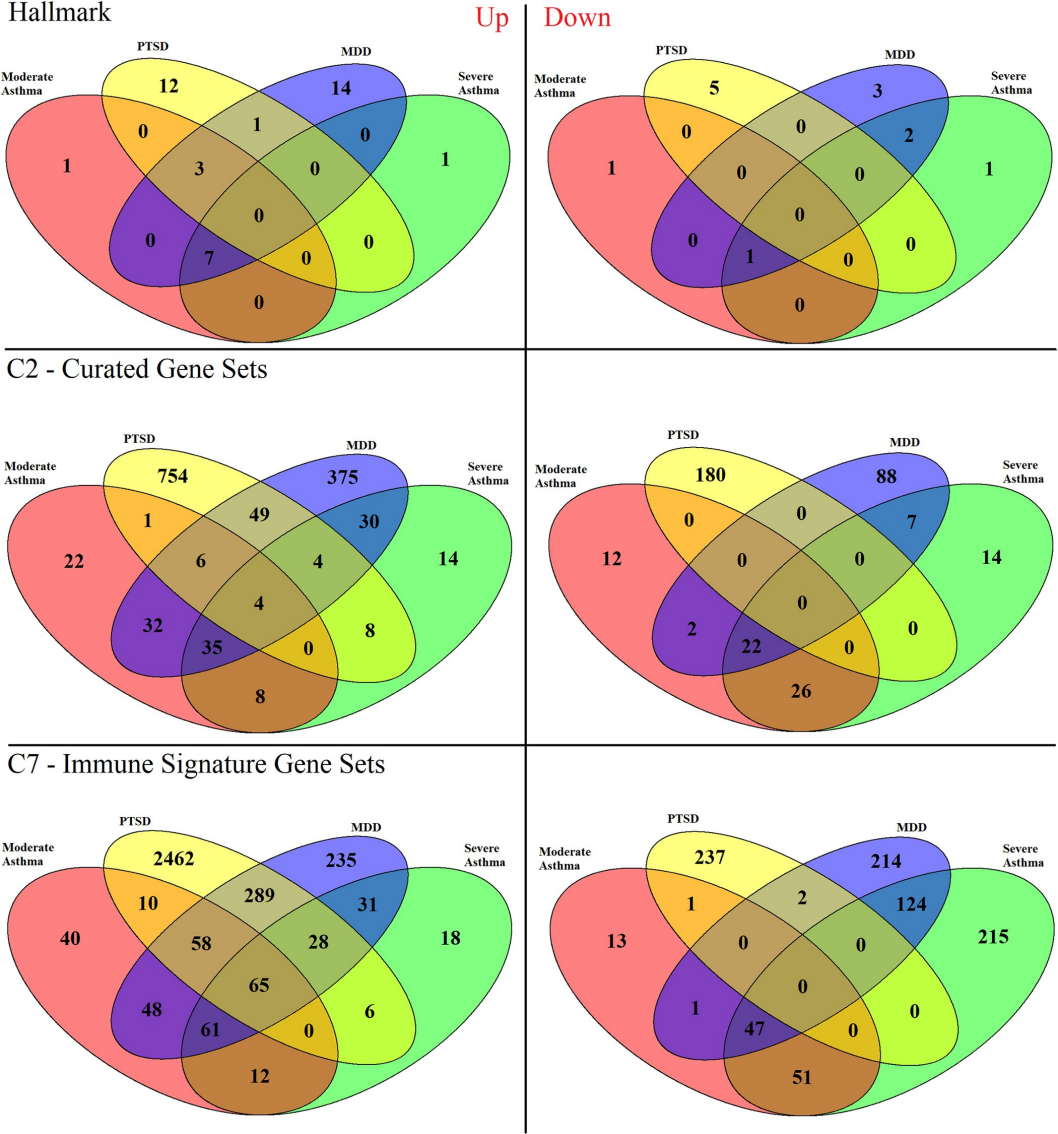
Gene set enrichment analysis. (GSEA) showing significantly (adjusted P-value < 0.05) modified pathways between all 4 exploration datasets. Labels identify the gene sets being compared to and in which direction (left = upregulated, right = downregulated).

To detect the biological effect of more nuanced changes in all disease groups, Gene Set Enrichment Analysis (GSEA) was performed. GSEA compared expression of selected lists of genes (here termed “gene sets”) between diseased and healthy subjects in each dataset (Fig 4). Gene sets from the Hallmark, C2, and C7 collections were compared against. Hallmark gene sets are sets of genes that comprise 50 of the best studied signaling pathways in the body. The C2 gene sets, or curated gene sets, in addition to the well understood and mapped ‘KEGG pathways’, include other sets of genes found previously to be differentially expressed in literature. C7 gene sets are immune signature gene sets found previously to be differentially expressed in literature.

No Hallmark gene sets were enriched in the same direction between all 4 datasets. 3 C2 gene sets were found to be upregulated in all 4 groups: REN_ALVEOLAR_RHABDOMYOSARCOMA_DN, JISON_SICKLE_CELL_DISEASE_UP, TAKEDA_TARGETS_OF_NUP98_HOXA9_FUSION_8D_DN, and REACTOME_NEUTROPHIL_DEGRANULATION. No C2 gene sets were commonly downregulated in all 4 groups. 65 C7 gene sets were commonly upregulated in all 4 groups, but nothing was commonly downregulated in all 4 of those groups.

### Validation of differentially expressed genes in independent transcriptomic datasets

To challenge these findings, the ‘validation’ datasets for each of: MDD, PTSD, severe asthma, and moderate asthma underwent DE analysis with limma. No genes were found to be significantly regulated (adjusted P-value < 0.05) in the same directions for all 4 sets as no individual genes were significantly differentially expressed in the MDD validation dataset. 2 genes were validated as upregulated in PTSD and severe asthma: *STX8* (Adjusted p-values in PTSD exploration, PTSD validation, severe asthma exploration, severe asthma validation were: 1.6E-3, 1.8E-2, 2.6E-3, 3.7E-4) and *ARHGAP24* (1.4E-2, 1.6E-2, 3.9E-2, 3.2E-2). Commonly downregulated to PTSD and severe asthma were *ORMDL3* (2.2E-2, 1.9E-3, 2.7E-3, 3.9E-3), *PTP4A3* (2.6E-3, 2.3E-2, 4.5E-3, 5.2E-3), *SHISA4* (1.1E-2, 4.4E-2, 9.8E-3, 6.2E-3), and *TPPP3* (2.2E-2, 1.1E-2, 3.1E-3, 2.7E-2). No differentially expressed genes were validated between PTSD and moderate asthma in either direction, however. 582 genes were validated as significantly downregulated between moderate and severe asthma while no upregulated genes could be validated.

### Validation of regulated pathways in independent transcriptomic datasets

The same datasets used to validate differentially expressed genes were used to validate gene sets and pathways identified as being commonly regulated in either direction in the exploration datasets. Interestingly, despite no genes being significantly differentially expressed in MDD patients vs healthy controls in the validation dataset, there were pathways identified as being significantly altered in severe asthma patients as compared to their corresponding controls (Table 1).

**Table 1:** Directionally validated pathway comparisons in the Hallmark, C2, and C7 collections.

Direction	Comparison	Enriched Gene Set	Adjusted P-Values
Up	MDD and Severe Asthma C7	GSE4748_CYANOBACTERIUM_LPSLIKE_VS_LPS_AND_CYANOBACTERIUM_LPSLIKE_STIM_DC_3H_DN	MDD1 - 1.58e-11, MDD2 - 1.30e-2, S.Asthma1 - 5.49e-14, S.Asthma2 - 3.42e-12
		GSE34205_HEALTHY_VS_RSV_INF_INFANT_PBMC_DN	MDD1 - 7.69e-12, MDD2 - 1.16e-2, S.Asthma1 - 1.79e-10, S.Asthma2 - 6.29e-10
Down	MDD and Severe Asthma C7	GSE22886_NAIVE_BCELL_VS_NEUTROPHIL_UP	MDD1 - 1.30e-10, MDD2 - 2.61e-3, S.Asthma1 - 4.41e-7, S.Asthma2 - 2.27e-9
		GSE34205_HEALTHY_VS_FLU_INF_INFANT_PBMC_UP	MDD1 - 4.69e-3, MDD2 - 2.26e-2, S.Asthma1 - 4.47e-3, S.Asthma2 - 9.99e-6
		GSE22886_NEUTROPHIL_VS_MONOCYTE_DN	MDD1 - 3.37e-2, MDD2 - 7.46e-8, S.Asthma1 - 3.74e-2, S.Asthma2 - 3.40e-3
Down	MDD and Severe Asthma C2	JISON_SICKLE_CELL_DISEASE_DN	MDD1 - 1.37e-8, MDD2 - 4.42e-2, S.Asthma1 - 4.44e-3, S.Asthma2 - 9.13e-5

Directionally validated pathway comparisons in the Hallmark, C2, and C7 collections, following GSEA excluding comparison between severe and moderate asthma.

As may be expected, many pathways were found to be commonly modified between moderate and severe asthma when comparing against the C2 and C7 gene sets and although they are not the focus of this study on comorbidity, can be found listed in supplementary information (S1 Table in S1 File). Barcode plots showing a more detailed cross-section of gene expression from the sets in Table 1 can be found in supplementary information (S1-S6 Figs in S1 File).

**Fig 5:**
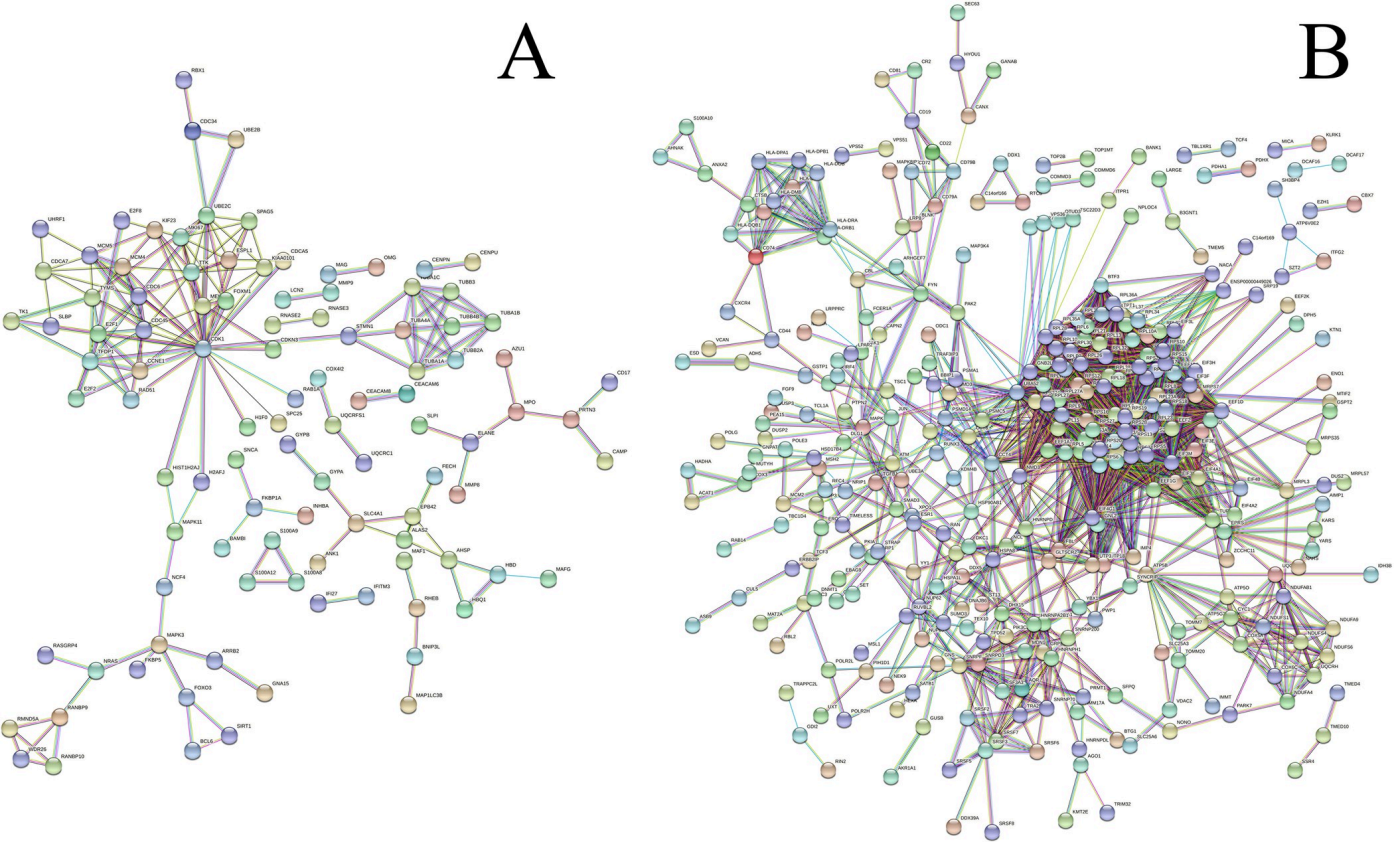
STRING analysis for proteins coded by genes. Found A) enriched in the peripheral whole blood of both MDD and severe asthma patients compared to healthy subjects, and B), enriched in healthy subjects compared to both MDD and severe asthma patients. Only high confidence interactions between proteins are shown, and proteins that are not involved in a high confidence interaction do not appear.

Finally, we pooled all genes from each significantly differentially expressed set common to MDD and severe asthma (Table 1) and performed a STRING cluster analysis for proteins to determine if any other functional networks emerged. Two networks were examined, grouping genes enriched in both MDD and severe asthma compared to healthy subjects, as well as genes enriched in healthy subjects compared to MDD and severe asthma (Fig 5). Among many other associations, STRING analysis found that proteins encoded by the disease-enriched genes of the “GSE4748_CYANOBACTERIUM_LPSLIKE_VS_LPS_AND_CYANOBACTERIUM_LPSLIKE_STIM_DC_3H_DN,” and “GSE34205_HEALTHY_VS_RSV_INF_INFANT_PBMC_DN” gene sets have been previously identified in literature in various roles - including modulation of immune function, cancer involvement, and more (Fig 5A). The complete list of functional annotations can be found in S1 and S2 Data.

Likewise, proteins encoded by the healthy subject-enriched genes of the “GSE22886_NAIVE_BCELL_VS_NEUTROPHIL_UP,” “GSE34205_HEALTHY_VS_FLU_INF_INFANT_PBMC_UP,” “GSE22886_NEUTROPHIL_VS_MONOCYTE_DN,” and “JISON_SICKLE_CELL_DISEASE_DN” gene sets have been shown involved in several functional enrichments including several facets of ribosome regulation, and MHC class II activity. The complete list of functional annotations can be found in S1 and S2 Data.

## Discussion

While it is widely accepted that psychosocial factors affect asthma pathobiology in children and adults, there is little understanding of potential common biological pathways underlying comorbidity between asthma and mental health disorders. Previous reports based on GWAS studies were focusing on determination of shared genetic traits between Asthma, MDD and PTSD or on circulating levels of specific inflammatory cytokines to explore potential shared pathophysiology of these disorders. In an attempt to provide further insight into the comorbidity of these conditions and to identify target pathways for further investigation, we utilized publicly available data to assess similarities between asthma, MDD and PTSD at the transcriptomic level.

As expected, asthma, MDD and PTSD were associated with many differentially expressed genes and gene sets, and, in comparing exploration cohorts, a number of these genes and gene sets were significantly regulated in the same direction in all diseases. Upon validation, commonalities in transcriptomic changes were restricted to comparisons between severe asthma and MDD or PTSD.

In keeping with literature indicating a close association with regards to comorbidity and reciprocal enhancement of symptom severity [17, 18], our cross-disease comparisons found the greatest transcriptomic level similarities between severe asthma and PTSD.

With regard to commonly differentially expressed genes we found *ORMDL3* to be downregulated in the blood of both PTSD and severe asthma subjects. *ORMDL3* codes for a protein called “ORMDL sphingolipid biosynthesis regulator 3” which resides in the endoplasmic reticulum and is a regulator of sphingolipid synthesis [30]. ORMDL3 requires precise expression to function correctly - under normal conditions it inhibits the rate limiting enzyme of sphingolipid biosynthesis, serine palmitoyl transferase (SPT) [30]. Downstream of uninhibited SPT activity, ceramide - the central sphingolipid metabolite - is produced and transported to the golgi [31]. Therefore, a knockdown of ORMDL3 can result in an abundance of ceramide [32]. When slightly overexpressed, ORMDL3 leads to a dearth of ceramide, however, when highly overexpressed, ORMDL3 increases ceramide biosynthesis through the alternate, recycling/salvage pathway [33, 34].

Numerous GWAS have identified ORMDL3 as a potential susceptibility gene for asthma and polymorphisms controlling ORMDL3 expression have been associated with both asthma occurrence and exacerbation [34–41].

However, the mechanistic contribution of ORMDL3 to the pathogenesis of asthma remains unclear and experimental evidence suggests the relationship between ORMDL3 and asthma is complex. Studies in animal models of allergic airway inflammation have indicated that overexpression of ORMDL3 leads to increased ceramide levels and the accompanying ER stress leads to characteristic features of asthma including increased mucus production, an exacerbated inflammatory response, and airway hyperresponsiveness. Correspondingly, downregulation of ORMDL3 expression, and decreased ceramide levels, were demonstrated to significantly ameliorate asthmatic symptoms in a mouse model [33, 42–46]. Furthermore, the expression of ORMDL3 in eosinophils seems to play a role in recruitment, attachment and activation of eosinophils in asthma [47]. However, seemingly conflicting evidence suggests that decreased expression of ORMDL3 can also promote asthma symptoms. Selective knockdown of ORMDL3 in lung epithelial cells leads to airway hyperresponsiveness [48], while downregulation of ORMDL3 in mast cells, cells key to asthma pathogenesis, enhances antigen mediated expression of proinflammatory cytokines and production of prostaglandin D2 and promotes mast cell driven inflammation in vivo [49].

While, to our knowledge there have been no studies associating ORMDL3 and PTSD, ceramide is a precursor for complex sphingolipids that are highly abundant in neural cellular membranes and are regulators of brain homeostasis [50]. Ceramide has also been shown to promote stress-induced depression-like behavior in mice, and intervention with drugs that reduce hippocampal ceramide (amitriptyline and fluoxetine) rescued those behaviours [51, 52].

Upregulated in the blood of severe asthma and PTSD subjects were mRNA encoding Syntaxin 8 (STX8), and Rho GTPase Activating Protein 24 (ARHGAP24). STX8 is a t-SNARE protein (target soluble N-ethylmaleimide-sensitive factor attachment protein receptor) involved in diverse vesicle docking and membrane fusion events. STX8 has been demonstrated to regulate the function of receptors and ion channels, including TrkA and CFTR. The TrkA receptor is transported from the golgi to the plasma membrane by STX8, a process which with nerve growth factor (NGF) stimulation promotes downstream TrkA signaling [53]. Interestingly, higher levels of TrkA expression have been identified in patients with allergic asthma [54], and although its role in asthma has not been fully elucidated, there are several proposed mechanisms by which neurotrophin signaling exacerbates asthma [55]. Some evidence suggests neurotrophin signaling may modulate airway hyperactivity and bronchoconstrictor release, enhancement of airway contractility, as well as airway remodeling [55–57]. TrkA has also been previously implicated in PTSD, as NGF signaling via TrkA alleviated stress induced PTSD-like symptoms in mice [58]. In contrast to enhancing TrkA signaling, STX8 also interacts with CFTR to inhibit function and trafficking to the cell surface [59]. CFTR is largely studied in relation to cystic fibrosis, however, impaired function of this ion channel has been associated with more severe or difficult to treat asthma [60–62]. While to our knowledge there has been no suggested relationship between CFTR and PTSD, the ion channel is expressed throughout the central nervous system [63].

ARHGAP24 converts the Rac-type GTPase into its inactive GDP-bound state which, downstream of Rho, suppresses actin remodelling [64]. Increased activation of RhoA/Rho-kinase is associated with airway hyper-responsiveness and smooth muscle contraction in asthma [65]. Cerebral RhoA activation is known to enhance fear memory which may have implications for PTSD [66]. So, in both asthma and PTSD, increased Rho activity is associated with increased pathology. It is curious then, that we find an inhibitor of its downstream activity differentially overexpressed in the blood of the diseased subjects. The reason for this would have to be elucidated by further research.

Other genes commonly downregulated in severe asthma and PTSD were Protein Tyrosine Phosphatase 4A3 (PTP4A3), known for its role in stimulating progression from G1 to S phase in mitosis [67]; Shisa Family Member 4 (SHISA4), a transmembrane scaffold/adaptor protein [68]; and Tubulin Polymerization Promoting Protein Family Member 3 (TPPP3), a regulator of microtubule dynamics [69]. To our knowledge, none of these proteins have previously been associated with asthma or PTSD and their identification here may warrant further investigation.

Neither ORMDL3, STX8, nor ARHGAP24 are discussed by Bigler *et al*., (2017) [70] in relation to the asthma datasets; nor are they identified in the PTSD dataset by Rusch *et al*., (2019) [71]. ARHGAP24 is discussed briefly in regards to PTSD in the validation data set, (Kuan *et al*., 2017) [72] as being a member of the PTSD-associated actin cytoskeleton pathway.

One of the gene sets “GSE34205_HEALTHY_VS_RSV_INF_INFANT_PBMC_DN’’ refers to a list of genes found to be more highly expressed in peripheral blood mononuclear cells (PBMC) of infants with RSV (Respiratory syncytial virus) bronchiolitis [69] when compared to those of healthy subjects. We also found that “GSE34205_HEALTHY_VS_FLU_INF_INFANT_PBMC_UP,” a list of genes with decreased in expression infants with acute influenza compared to PBMCs of healthy subjects, was downregulated in both MDD and severe asthma [73]. These 2 congruent pieces of evidence suggest that the immune signature to respiratory infection in infants is similar to the immune signature of both asthma and MDD whole blood. In human airway epithelial cells Ioannidis *et al*., (2012) [73] found that comparing both influenza and RSV treatment to control exhibited DE reminiscent of a type I interferon immune signature and genes downstream of IFN-α/β were expressed abundantly in infected cells. Type I interferon signaling is known to be a contributing factor in some cases of both depression and asthma [74–77].

Two additional gene sets we found downregulated in both MDD and severe asthma: “GSE22886_NAIVE_BCELL_VS_NEUTROPHIL_UP,” and “GSE22886_NEUTROPHIL_VS_MONOCYTE_DN’’ were both compiled by Abbas *et al*., (2005) [78] to identify patterns in immune cell-specific expression in order to identify states of activation. The gene sets we identified as being underexpressed in MDD and severe asthma can be congruently explained by a reduction of neutrophil specific gene expression, or by an increase in naive B-cell and monocyte specific gene expression. The latter is perhaps more likely as neutrophils have been demonstrated to be activated in patients with MDD and asthma [79, 80]. Furthermore, B cell homeostasis is altered in individuals with MDD and B cells play a crucial role in regulating the hyperactivity of airways in asthma [81–84]. Likewise, there is generally increased activity and larger numbers of monocytes in MDD and asthma compared to healthy subjects [85–88]. This highlights the possibility of enhanced B cell and monocyte activity playing a key role in comorbid asthma and MDD.

JISON_SICKLE_CELL_DISEASE_DN, found downregulated in the blood for both MDD and severe asthma, are genes previously found to be downregulated in peripheral blood mononuclear cells (PBMCs) in sickle-cell disease patients compared with non-diseased counterparts. Asthma is common in children with sickle cell disease and this comorbidity is becoming increasingly well documented [89]. In sickle cell, nitric oxide consumption mediated by plasma hemoglobin, ischemia-reperfusion injury, and the generation of free radicals activate an inflammatory stress response [89]. Jison *et al*., (2004) [90], who discovered the gene set, found many of the genes differentially expressed within PBMCs were linked to inflammatory stress as well. To find these same genes underexpressed in two comorbid conditions suggests that the inflammatory stress response itself could be a driver behind comorbidity for sickle cell disease, MDD, and severe asthma.

The STRING analysis for proteins translated from the individual genes in the gene sets commonly regulated between MDD and severe asthma show that the genes upregulated in each of these diseases have several functional associations. By combining curated gene sets enriched in both diseases we gave the string analysis a more complete picture of all the systems that may be modified downstream of these blood transcriptional changes. In addition to basic biological processes, cellular compartments, molecular functions, and pathways, several smaller literature-backed gene sets were found in common. Examining the top 5 in descending order of strength our genes enriched in MDD and severe asthma, we observed matches to biomarkers for severe influenza infection (Adj. P-value = 2.2E-5) [91], genes associated with arthritis (Adj. P-value = 1.5E-3) [92], respiratory distress syndrome phenotypes (Adj. P-value = 1.2E-2) [93], lung epithelial function in sepsis (Adj. P-value = 5.6E-6) [94], and myocardial infarction and neutrophil degranulation (Adj. P-value = 2.6E-4) [95]. Looking at the top 5 for genes enriched in healthy subjects compared to MDD and severe asthma we identified many matches associated with ribosomal regulation, and to a lesser extent immune function and anemia (Adj. P-values = 3.1E-9, 2.0E-4, 2.0E-4, 1.3E-3, 6.5E-3) [96–100]. This could suggest that there is less ribosomal regulation in MDD and severe asthma, and further suggests that immune involvement could drive the relationship between these disorders.

Despite MDD being a major comorbidity in PTSD, and 440 immune signature gene sets commonly upregulated between the exploration datasets, no genes or gene sets were validated in this study when comparing MDD and PTSD. However, the neurobiology of the link between PTSD and MDD is unclear and it is entirely feasible that similarities in gene expression between the disorders is restricted to the CNS and are undetectable in the blood.

It is notable that there were no validated genes or gene sets in common between mild/moderate asthma and either of the mental health disorders. This finding is consistent with the phenomenon that mental health disorders such as PTSD and MDD are correlated with more severe disease outcomes [101]. It may be that activation of specific genes or pathways that are involved in MDD or PTSD are also factors that contribute to the development of more severe asthma. In this regard, there is evidence to suggest that antidepressant treatment improved asthma symptoms in severe but not mild asthmatics with co-morbid depression.

Overall, with six parallel DGE analyses and GSEA on whole blood gene expression, we identified genes and gene set expression that potentially links severe asthma to both PTSD and MDD. The gene sets commonly regulated between asthma and MDD, support previously suggested links between inflammation related immune factors and the two disorders [7]. Epidemiological evidence indicating that PTSD has a stronger association with asthma than other chronic inflammatory diseases [102, 103] suggests that the relationship is driven by more than common immune factors. Here we identify 6 genes (2 upregulated in disease and 4 downregulated) being differentially expressed in both PTSD and asthma. Of particular note, our results identify mechanisms involving ceramide biosynthesis and SNARE regulated signaling pathways as potential targets for future research aimed at understanding both the relationship between PTSD and asthma and the pathophysiology of the individual disorders.

## Methods

### Obtaining and preprocessing datasets

Data were downloaded from the Gene Expression Omnibus (GEO) repository and preprocessed using the methods described by the respective authors associated with each dataset (Table 2). Specific blood RNA datasets were chosen over others on GEO due to there being among the few datasets on GEO that met the specific criteria of whole blood (rather than PBMCs, or biopsy), the specific diseases in question, and focused on mRNA (rather than total RNA or miRNA). Any remaining appropriate datasets on GEO were on different platforms. We decided against pooling these datasets since attempts to correct for technical variation forces data modification that can confound and obscure the true biological variation of interest, and increase the likelihood of generating erroneous results. Therefore, we preferred to select the largest available datasets that did not require pooling for a classic exploration and validation analysis.

**Table 2:** List of datasets used in this paper.

GSE #	Platform (GPL)	Source and Species	Normalization Method	Purpose	Associated Publication	# Samples	# Genes / Variants
GSE81761	GPL570 (Array)	Human whole blood mRNA	RMA*	PTSD Exploration	Rusch *et al*., 2019	27 - PTSD, 39 - No PTSD	44,134
GSE97356	GPL11154 (RNAseq)	Human whole blood mRNA	TMM	PTSD Validation	Kuan *et al*., 2017	82 - PTSD, 201 - No PTSD	15,112
GSE98793	GPL570 (Array)	Human whole blood RNA	RMA	MDD Exploration	Leday *et al*., 2018	64 - MDD, 32 - No MDD	44,134
GSE19738	GPL6848 (Array)	Human whole blood RNA	Quantile	MDD Validation	Spijker *et al*., 2010	33 - MDD, 34 - No MDD	12,816
GSE69683	GPL13158 (Array)	Human whole blood RNA	RMA	Asthma Exploration and Validation	Bigler *et al*., 2017	After Split:	41,791
						Exploration: 58 - Healthy, 58 - Moderate, 216 - Severe.	
						Validation: 28 - Healthy, 20 - Moderate, 128 - Severe	

List of datasets used in this paper with a description of data type, preprocessing, number of genes and gene variants remaining in the dataset following preprocessing, and associated publications.

* Robust multichip average (RMA) normalization.

Rusch *et al*., (2019) [71] (preprocessed and raw data available at: https://www.ncbi.nlm.nih.gov/geo/query/acc.cgi?acc=GSE81761) measured blood mRNA military service members, with and without PTSD. Only samples from the first time-point collection, rather than the follow up collection, were selected for analysis. Other information collected on the subjects included sex (63 male, 3 female), age (22-49), and race. Kuan *et al*., (2017) [72] (preprocessed and raw data available at: https://www.ncbi.nlm.nih.gov/geo/query/acc.cgi?acc=GSE97356) measured blood mRNA in World Trade Center responders with PTSD currently, never, and in the past. Samples collected from subjects who never had PTSD or had PTSD at the time of the collection were selected for further analysis. No other sample information was supplied with the dataset. Leday *et al*., (2018) [104] (preprocessed and raw data available at: https://www.ncbi.nlm.nih.gov/geo/query/acc.cgi?acc=GSE98793) pooled human blood mRNA data from two depression studies: the “Janssen–Brain Resource Company “study, and the “GlaxoSmithKline–High-Throughput Disease-specific target Identification Program” study into subjects with MDD, and without. Batch 1 and batch 2 were originally found to generate distinct groups in principal component analysis (PCA), and were batch corrected with the ‘removeBatchEffect’ function in limma package (Ritchie *et al*., 2015 [105]) in R. This dataset contained additional information, such as including gender (144 female, 48 male), age (31-72), and anxiety status (128 no, 64 yes). Spijker *et al*., (2010) [106] (preprocessed and raw data available at: https://www.ncbi.nlm.nih.gov/geo/query/acc.cgi?acc=GSE19738) collected blood from subjects with and without MDD prior to and following stimulation with lipopolysaccharide (LPS), data which we excluded. Additional information in the dataset was age (21-63), gender (41 female, 26 male), and smoking status (20 non-smoking, 18 quit smoking, 29 smoking). The Unbiased Biomarkers for the Prediction of Respiratory Disease Outcomes (U-BIOPRED) study dataset (Bigler *et al*., 2017) [70] (preprocessed and raw data available at: https://www.ncbi.nlm.nih.gov/geo/query/acc.cgi?acc=GSE69683) measured blood mRNA in subjects with moderate (lung function tests are 60-80% of expected value), severe (lung function tests are <60% of expected value), and no asthma. The dataset also contained information on gender of the patients (275 female, 223 male), smoking or non-smoking (410 non-smoking, 88 smoking). We randomly divided this dataset into an exploration and a validation cohort at a 2:1 ratio. Low expressed genes were filtered out prior to trimmed mean of M-values (TMM) normalization of the RNAseq dataset as it is more sensitive due to its single nucleotide resolution [107, 108]. This was performed using the edgeR packages ‘filterByExpr’ function [109]. Data on race was only available in the Rusch *et al*., (2019), and Bigler *et al*., (2017) datasets and both studies had predominantly white caucasian participants (66% and 90%, respectively).

Seeing as not all datasets contained the same background information on their respective subjects, and because the purpose of this study was to detect commonalities between comorbid diseases that may exist robustly in a particular disease regardless of other variables, demographic information such as age, race, gender, and smoking status was not taken into consideration.

Principal component analysis (PCA) was done in base R and visualized using ggplot2 [110]. Venn diagrams were generated using the VennDiagram R package [111].

### Differential gene expression

Each dataset, including the split asthma datasets for both severe and moderate asthma, underwent differential gene expression analysis individually, comparing their disease to the respective control group (the non-disease group) from the same study. Analysis was performed using the limma package with multiple hypothesis correction and Benjamini-Hochberg FDR applied. Genes were considered to be differentially expressed with an adjusted p-value < 0.05 and |FC| ≥ 1.5.

#### Gene set enrichment analysis

3 MSigDB collections of gene sets (v7.4) were downloaded from the GSEA website (https://www.gsea-msigdb.org/gsea/msigdb/index.jsp): Hallmark - well-defined biological states or processes, C2 - curated gene sets from PubMed publications and online pathway databases (including KEGG), and C7 - immunologic signature gene sets representative of immune and cell states.

Fold change values generated by the differential expression analysis of diseased subjects vs healthy subjects were compared to each of the 3 collections via their entrez gene IDs using the Gage package in R [112]. Gage uses the differential expression output of all genes, not just those with significant fold change or p-value. Barcode plots were generated using barcodeplot() function (limma package). Volcano plots were generated using the R package ‘EnhancedVolcano’ [113].

## Supporting information

S1 File (DOCX)

S1 Data (TSV)

S2 Data (TSV)
