# Correction: Severe, but not moderate asthmatics share blood transcriptomic changes with post-traumatic stress disorder and depression

**DOI:** 10.1371/journal.pone.0318537

**Published:** 2025-01-27

**Authors:** The PLOS One Staff

## Notice of republication

This article was republished on January 16, 2025, to correct an error in the author list: Sandor Haas-Neill was erroneously displayed as Sandor Haas-Neil. The publisher apologizes for the errors. Please download this article again to view the correct version. The originally published, uncorrected article and the republished, corrected articles are provided here for reference.

## Supporting information

S1 File Originally published, uncorrected article. (PDF)

S2 File Republished, corrected article. (PDF)
