# Supplementary material for: Severe, but not moderate asthmatics share blood transcriptomic changes with post-traumatic stress disorder and depression
Source: PLoS One. 2022 Oct 7;17(10):e0275864. doi: 10.1371/journal.pone.0275864 (PMC9543640; doi:10.1371/journal.pone.0275864)
Supplement: S1 File — (DOCX) [file pone.0275864.s001.docx]

**Supplementary Information**

**S1 Table. Pathways commonly differentially regulated between severe and moderate asthma**

|  |  | **Q. Explore S** | **Q. Validate S** | **Q. Explore M** | **Q. Validate M** |
| --- | --- | --- | --- | --- | --- |
| C2 Up |  |  |  |  |  |
|  | TAKEDA_TARGETS_OF_NUP98_HOXA9_FUSION_10D_DN | 3.65E-004 | 1.06E-003 | 1.79E-002 | 1.80E-002 |
|  | ALTEMEIER_RESPONSE_TO_LPS_WITH_MECHANICAL_VENTILATION | 5.54E-003 | 4.43E-006 | 2.08E-004 | 4.84E-002 |
|  | VERHAAK_AML_WITH_NPM1_MUTATED_UP | 8.48E-007 | 1.42E-008 | 1.36E-006 | 8.29E-004 |
|  | SMIRNOV_CIRCULATING_ENDOTHELIOCYTES_IN_CANCER_UP | 1.99E-006 | 1.72E-003 | 1.00E-004 | 1.75E-002 |
|  | REN_ALVEOLAR_RHABDOMYOSARCOMA_DN | 3.09E-003 | 3.23E-002 | 9.36E-003 | 2.12E-002 |
|  | JISON_SICKLE_CELL_DISEASE_UP | 1.44E-003 | 1.15E-006 | 7.58E-005 | 1.35E-002 |
|  | REACTOME_NEUTROPHIL_DEGRANULATION | 7.23E-020 | 1.20E-013 | 3.05E-007 | 1.54E-003 |
| C7 Up |  |  |  |  |  |
|  | GSE10325_CD4_TCELL_VS_MYELOID_DN | 2.24E-005 | 2.24E-004 | 7.93E-007 | 1.61E-004 |
|  | GSE10325_BCELL_VS_MYELOID_DN | 3.83E-012 | 6.76E-010 | 1.15E-010 | 1.34E-003 |
|  | GSE10325_LUPUS_CD4_TCELL_VS_LUPUS_MYELOID_DN | 4.13E-013 | 1.03E-008 | 3.27E-016 | 2.30E-002 |
|  | GSE11057_NAIVE_CD4_VS_PBMC_CD4_TCELL_DN | 1.98E-004 | 2.12E-003 | 1.05E-005 | 8.84E-005 |
|  | GSE11057_CD4_EFF_MEM_VS_PBMC_DN | 2.22E-003 | 4.71E-003 | 4.06E-005 | 8.84E-005 |
|  | GSE11057_CD4_CENT_MEM_VS_PBMC_DN | 8.59E-006 | 3.92E-002 | 8.42E-009 | 3.65E-003 |
|  | GSE11057_PBMC_VS_MEM_CD4_TCELL_UP | 2.00E-005 | 6.57E-003 | 8.51E-011 | 2.81E-004 |
|  | GSE22886_NAIVE_TCELL_VS_DC_DN | 1.13E-002 | 3.63E-002 | 1.26E-002 | 2.26E-004 |
|  | GSE22886_NAIVE_TCELL_VS_MONOCYTE_DN | 4.13E-013 | 5.98E-010 | 5.20E-008 | 6.88E-004 |
|  | GSE22886_NAIVE_CD8_TCELL_VS_MONOCYTE_DN | 9.19E-012 | 7.10E-009 | 8.61E-008 | 2.50E-003 |
|  | GSE22886_NAIVE_CD4_TCELL_VS_MONOCYTE_DN | 4.99E-011 | 5.03E-011 | 1.35E-005 | 1.17E-004 |
|  | GSE24634_TREG_VS_TCONV_POST_DAY3_IL4_CONVERSION_DN | 5.67E-003 | 9.80E-003 | 4.45E-003 | 4.52E-003 |
|  | GSE24634_TREG_VS_TCONV_POST_DAY10_IL4_CONVERSION_DN | 6.69E-005 | 1.16E-003 | 2.71E-005 | 4.65E-003 |
|  | GSE24634_IL4_VS_CTRL_TREATED_NAIVE_CD4_TCELL_DAY10_DN | 5.34E-003 | 1.69E-004 | 1.59E-004 | 7.05E-003 |
|  | GSE29618_BCELL_VS_MONOCYTE_DN | 4.00E-009 | 1.50E-006 | 6.46E-006 | 1.50E-004 |
|  | GSE29618_BCELL_VS_MDC_DN | 1.67E-003 | 5.11E-003 | 2.63E-004 | 4.29E-003 |
|  | GSE29618_MONOCYTE_VS_PDC_UP | 8.75E-012 | 1.23E-009 | 2.20E-008 | 2.27E-003 |
|  | GSE29618_MONOCYTE_VS_MDC_UP | 1.72E-014 | 6.29E-011 | 3.69E-008 | 4.12E-004 |
|  | GSE29618_PDC_VS_MDC_DN | 6.04E-007 | 2.72E-006 | 1.83E-005 | 1.50E-002 |
|  | GSE29618_BCELL_VS_MONOCYTE_DAY7_FLU_VACCINE_DN | 4.91E-013 | 2.42E-007 | 9.08E-009 | 1.56E-002 |
|  | GSE29618_BCELL_VS_MDC_DAY7_FLU_VACCINE_DN | 2.37E-005 | 1.39E-003 | 4.42E-004 | 1.50E-002 |
|  | GSE29618_MONOCYTE_VS_PDC_DAY7_FLU_VACCINE_UP | 1.72E-014 | 4.18E-011 | 1.19E-009 | 1.82E-002 |
|  | GSE29618_MONOCYTE_VS_MDC_DAY7_FLU_VACCINE_UP | 2.15E-013 | 3.46E-010 | 5.79E-006 | 2.81E-004 |
|  | GSE29618_PDC_VS_MDC_DAY7_FLU_VACCINE_DN | 3.32E-006 | 3.48E-004 | 2.58E-006 | 6.48E-003 |
|  | GSE3982_EOSINOPHIL_VS_EFF_MEMORY_CD4_TCELL_UP | 3.59E-006 | 1.18E-004 | 3.00E-005 | 6.60E-004 |
|  | GSE3982_EOSINOPHIL_VS_CENT_MEMORY_CD4_TCELL_UP | 6.39E-008 | 1.51E-005 | 9.46E-006 | 2.67E-004 |
|  | GSE3982_EOSINOPHIL_VS_NKCELL_UP | 3.41E-004 | 1.18E-004 | 3.46E-004 | 6.88E-004 |
|  | GSE3982_BASOPHIL_VS_CENT_MEMORY_CD4_TCELL_UP | 2.31E-005 | 4.40E-006 | 2.46E-002 | 6.97E-003 |
|  | GSE34156_UNTREATED_VS_6H_TLR1_TLR2_LIGAND_TREATED_MONOCYTE_UP | 1.15E-007 | 6.63E-007 | 5.01E-007 | 2.50E-003 |
|  | GSE34156_UNTREATED_VS_24H_NOD2_LIGAND_TREATED_MONOCYTE_DN | 6.95E-006 | 3.01E-005 | 8.21E-007 | 1.90E-003 |
|  | SCHERER_PBMC_APSV_WETVAX_AGE_18_32YO_5_TO_7DY_UP | 1.36E-004 | 3.46E-010 | 6.67E-009 | 4.38E-003 |
|  | HOWARD_PBMC_INACT_MONOV_INFLUENZA_A_INDONESIA_05_2005_H5N1_AGE_19_39YO_AS03_ADJUVANT_VS_BUFFER_1DY_UP | 2.23E-011 | 4.50E-024 | 5.33E-017 | 2.06E-003 |
|  | NAKAYA_PBMC_FLUARIX_FLUVIRIN_AGE_18_50YO_CORRELATED_WITH_HAI_28DY_RESPONSE_AT_3DY_POSITIVE | 4.10E-015 | 1.14E-007 | 1.05E-009 | 1.03E-002 |
|  | GSE22886_NAIVE_TCELL_VS_NEUTROPHIL_DN | 1.72E-014 | 1.24E-012 | 1.31E-008 | 4.74E-002 |
|  | GSE6269_HEALTHY_VS_STAPH_PNEUMO_INF_PBMC_DN | 4.13E-013 | 1.49E-010 | 5.32E-006 | 3.51E-002 |
|  | GSE34156_TLR1_TLR2_LIGAND_VS_NOD2_AND_TLR1_TLR2_LIGAND_24H_TREATED_MONOCYTE_UP | 9.01E-009 | 3.16E-008 | 2.42E-008 | 6.97E-003 |
|  | GSE34156_NOD2_LIGAND_VS_TLR1_TLR2_LIGAND_6H_TREATED_MONOCYTE_DN | 6.29E-010 | 2.20E-007 | 1.57E-007 | 3.46E-002 |
|  | NAKAYA_PBMC_IMUVAC_MALE_AGE_14_27YO_1D_POSTBOOST_VS_0DY_PREIMM_TIV_UP | 1.36E-003 | 4.56E-004 | 2.25E-002 | 2.52E-002 |
|  | FLETCHER_PBMC_BCG_10W_INFANT_PPD_STIMULATED_VS_UNSTIMULATED_10W_DN | 9.57E-004 | 5.64E-003 | 1.85E-003 | 2.52E-002 |
|  | GSE3982_EOSINOPHIL_VS_DC_UP | 1.29E-002 | 1.36E-002 | 2.99E-002 | 2.52E-002 |
|  | GSE3982_EOSINOPHIL_VS_TH2_UP | 2.19E-002 | 3.79E-003 | 3.87E-002 | 7.22E-003 |
| C7 Down |  |  |  |  |  |
|  | GSE11057_CD4_CENT_MEM_VS_PBMC_UP | 3.20E-005 | 3.24E-012 | 2.63E-002 | 1.62E-004 |
|  | GSE11057_PBMC_VS_MEM_CD4_TCELL_DN | 1.05E-004 | 1.72E-010 | 1.27E-003 | 8.02E-003 |
|  | GSE22886_NAIVE_TCELL_VS_MONOCYTE_UP | 2.83E-014 | 1.91E-019 | 2.00E-004 | 4.00E-002 |
|  | GSE22886_NAIVE_CD8_TCELL_VS_DC_UP | 1.54E-003 | 4.35E-006 | 3.71E-002 | 2.22E-002 |
|  | GSE22886_NAIVE_CD8_TCELL_VS_MONOCYTE_UP | 4.24E-012 | 7.60E-011 | 1.68E-004 | 3.33E-002 |
|  | GSE22886_NAIVE_CD4_TCELL_VS_MONOCYTE_UP | 2.30E-009 | 1.95E-011 | 2.29E-004 | 2.22E-002 |

Pathways commonly differentially regulated between severe and moderate asthma and adjusted P (Q) values for: severe asthma (S) and moderate asthma (M) exploration and validation cohorts.

**
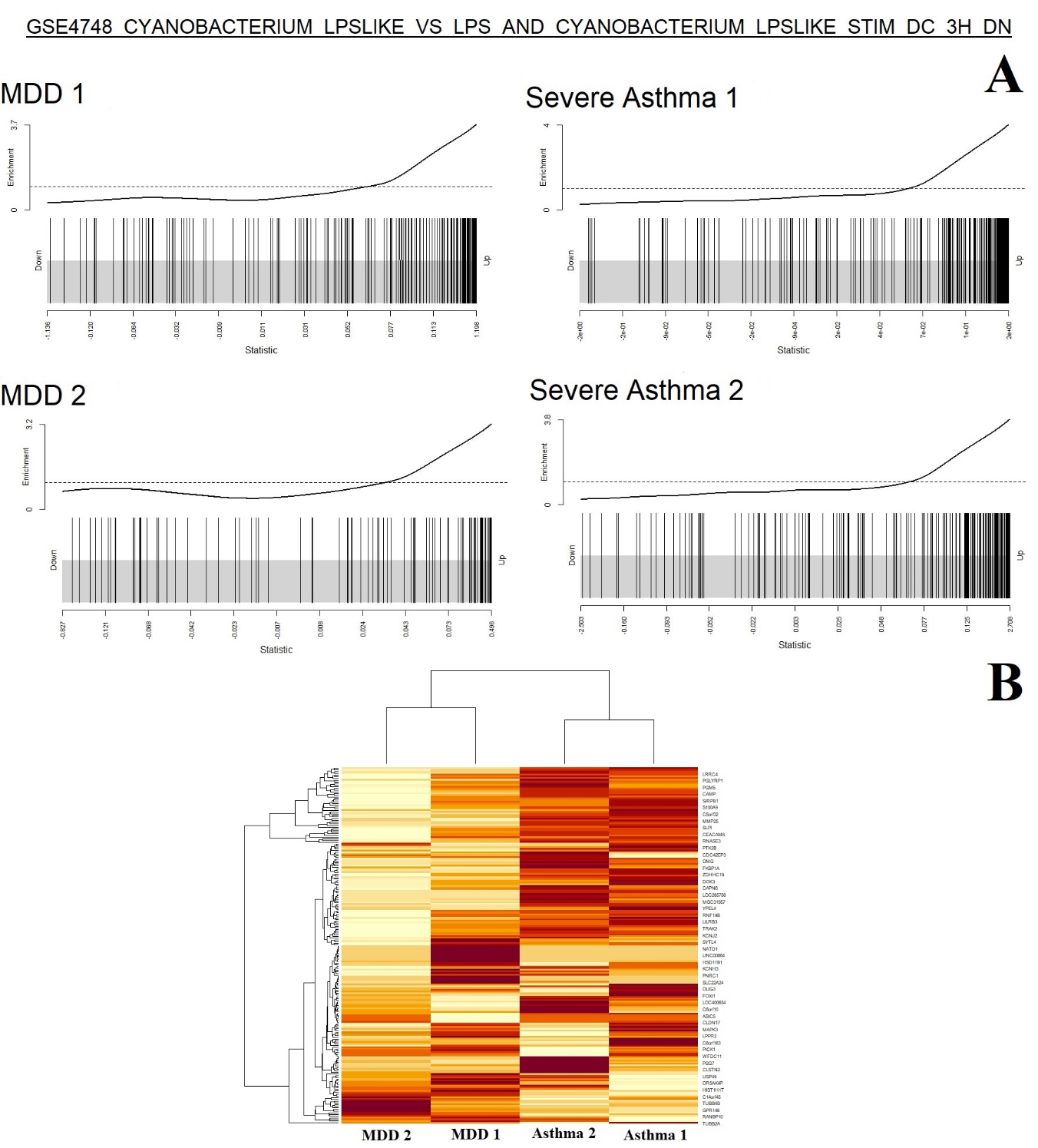
**

**S1 Figure.** Validated gene set expression in “GSE4748_CYANOBACTERIUM_LPSLIKE_VS_LPS_AND_CYANOBACTERIUM_LPSLIKE_STIM_DC_3H_DN.” A) Barcode plots showing cumulative individual genes in the gene set (as bars) for the exploration and validation datasets of MDD and severe asthma. Clusters of bars on one end represent individual genes that are differentially expressed in one direction or another within the gene set. B) A heatmap of differential expression of individual genes within the set for each of the exploration and validation MDD and severe asthma datasets.

**
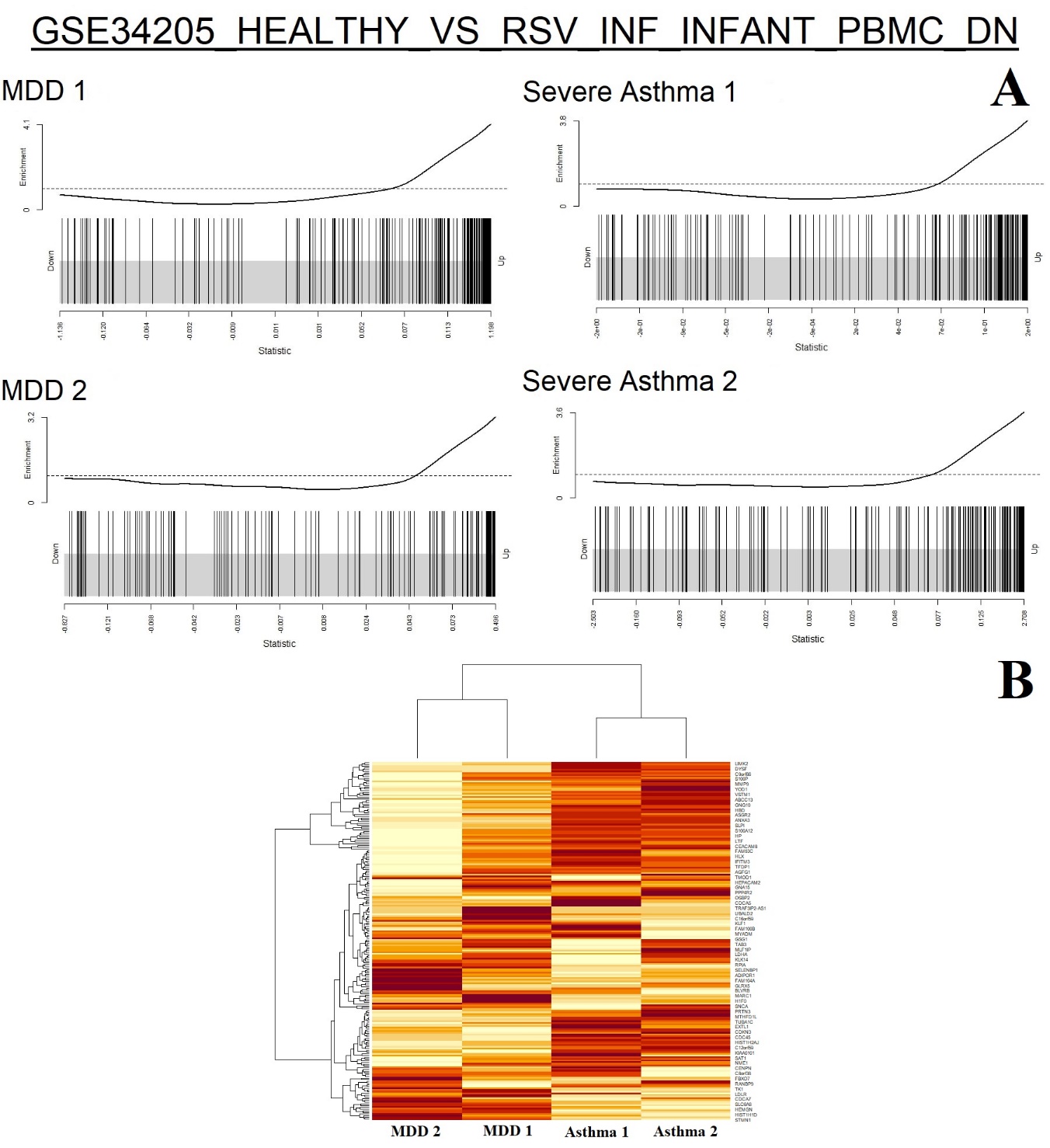
**

**S2 Figure.** Validated gene set expression in “GSE34205_HEALTHY_VS_RSV_INF_INFANT_PBMC_DN.” A) Barcode plots showing cumulative individual genes in the gene set (as bars) for the exploration and validation datasets of MDD and severe asthma. Clusters of bars on one end represent individual genes that are differentially expressed in one direction or another within the gene set. B) A heatmap of differential expression of individual genes within the set for each of the exploration and validation MDD and severe asthma datasets.

**
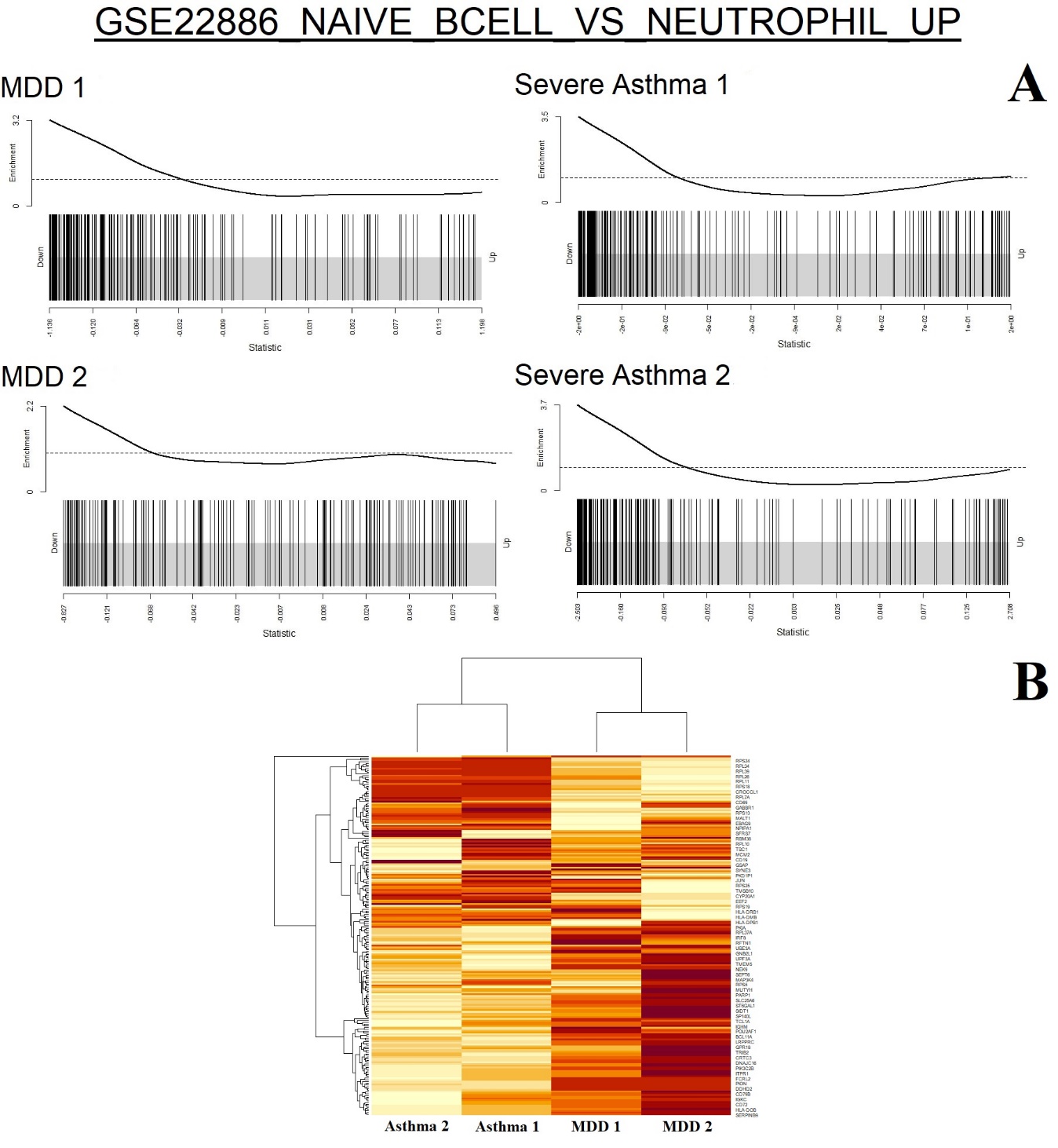
**

**S3 Figure.** Validated gene set expression in “GSE22886_NAIVE_BCELL_VS_NEUTROPHIL_UP.” A) Barcode plots showing cumulative individual genes in the gene set (as bars) for the exploration and validation datasets of MDD and severe asthma. Clusters of bars on one end represent individual genes that are differentially expressed in one direction or another within the gene set. B) A heatmap of differential expression of individual genes within the set for each of the exploration and validation MDD and severe asthma datasets.

**
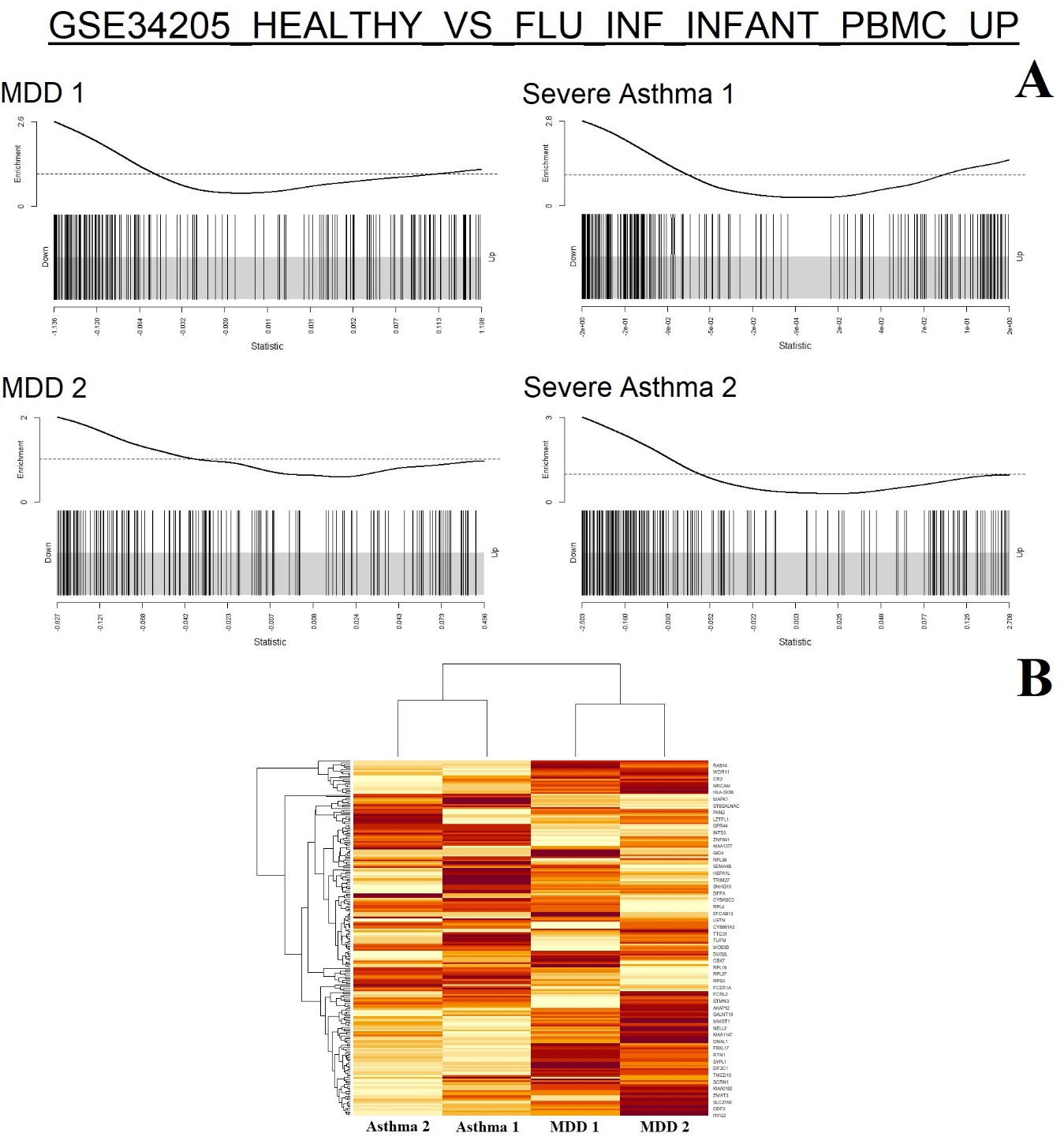
**

**S4 Figure.** Validated gene set expression in “GSE34205_HEALTHY_VS_FLU_INF_INFANT_PBMC_UP.” A) Barcode plots showing cumulative individual genes in the gene set (as bars) for the exploration and validation datasets of MDD and severe asthma. Clusters of bars on one end represent individual genes that are differentially expressed in one direction or another within the gene set. B) A heatmap of differential expression of individual genes within the set for each of the exploration and validation MDD and severe asthma datasets.

**
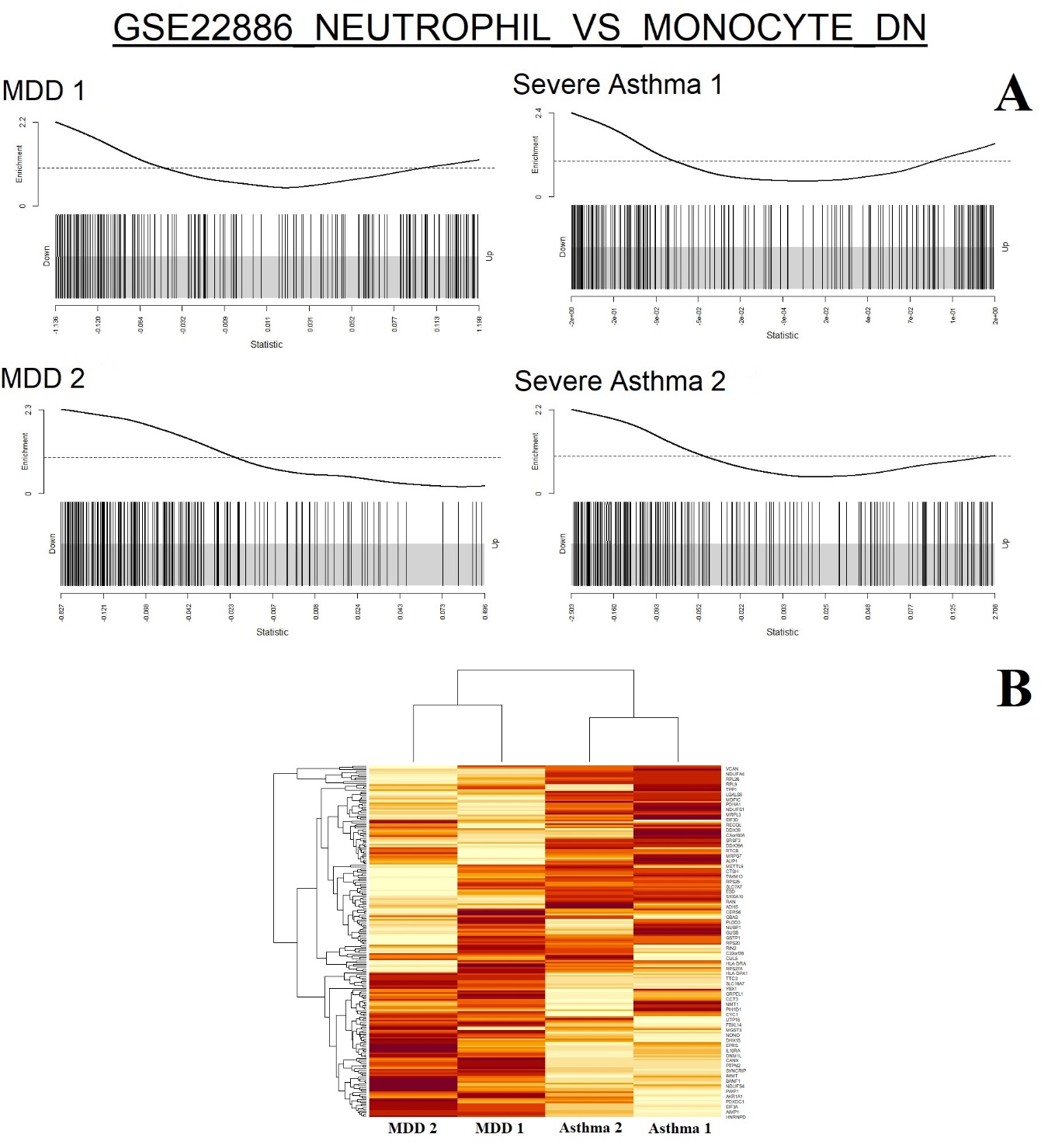
**

**S5 Figure.** Validated gene set expression in “GSE22886_NEUTROPHIL_VS_MONOCYTE_DN.” A) Barcode plots showing cumulative individual genes in the gene set (as bars) for the exploration and validation datasets of MDD and severe asthma. Clusters of bars on one end represent individual genes that are differentially expressed in one direction or another within the gene set. B) A heatmap of differential expression of individual genes within the set for each of the exploration and validation MDD and severe asthma datasets.


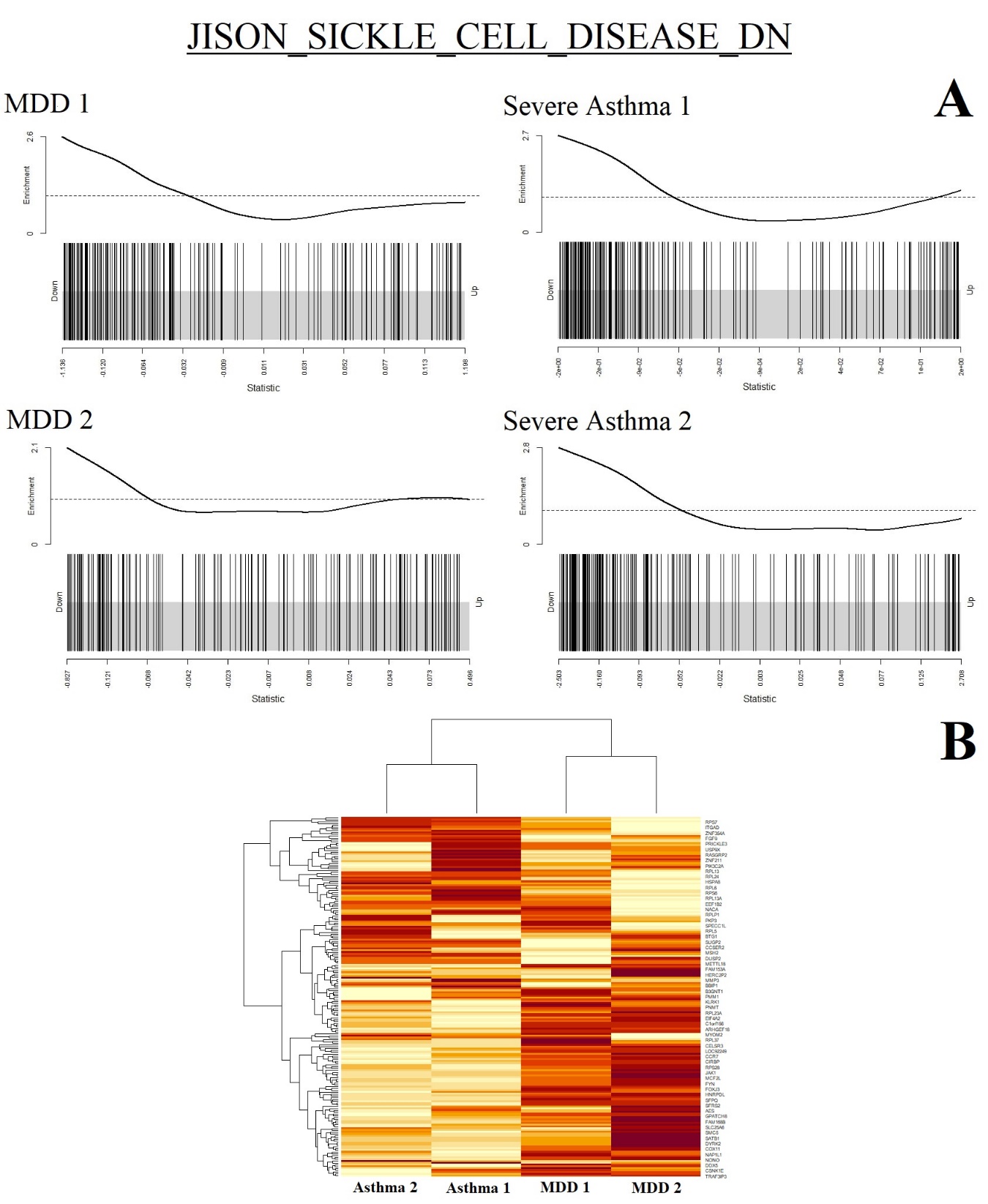


**S6 Figure.** Validated gene set expression in “JISON_SICKLE_CELL_DISEASE_DN.” A) Barcode plots showing cumulative individual genes in the gene set (as bars) for the exploration and validation datasets of MDD and severe asthma. Clusters of bars on one end represent individual genes that are differentially expressed in one direction or another within the gene set. B) A heatmap of differential expression of individual genes within the set for each of the exploration and validation MDD and severe asthma datasets.
