# Supplementary material for: Correction: Severe, but not moderate asthmatics share blood transcriptomic changes with post-traumatic stress disorder and depression
Source: PLoS One. 2025 Jan 27;20(1):e0318537. doi: 10.1371/journal.pone.0318537 (PMC11771866; doi:10.1371/journal.pone.0318537)
Supplement: S2 File — (PDF) [file pone.0318537.s002.pdf]

PLOS ONE

RESEARCH ARTICLE

Severe, but not moderate asthmatics share blood transcriptomic changes with post-traumatic stress disorder and depression

Sandor Haas-Neill[1], Anna Dvorkin-Gheva[2], Paul Forsythe[3]*

1 The Brain Body Institute, St. Joseph's Hospital, McMaster University, Hamilton, Ontario, Canada, **2** McMaster Immunology Research Centre, Department of Medicine, McMaster University, Hamilton, Ontario, Canada, **3** Alberta Respiratory Centre, Division of Pulmonary Medicine, Department of Medicine, University of Alberta, Edmonton, Alberta, Canada

* pforsyt1@ualberta.ca

Abstract

Asthma, an inflammatory disorder of the airways, is one of the most common chronic illnesses worldwide and is associated with significant morbidity. There is growing recognition of an association between asthma and mood disorders including post-traumatic stress disorder (PTSD) and major depressive disorder (MDD). Although there are several hypotheses regarding the relationship between asthma and mental health, there is little understanding of underlying mechanisms and causality. In the current study we utilized publicly available datasets of human blood mRNA collected from patients with severe and moderate asthma, MDD, and PTSD. We performed differential expression (DE) analysis and Gene Set Enrichment Analysis (GSEA) on diseased subjects against the healthy subjects from their respective datasets, compared the results between diseases, and validated DE genes and gene sets with 4 more independent datasets. Our analysis revealed that commonalities in blood transcriptomic changes were only found between the severe form of asthma and mood disorders. Gene expression commonly regulated in PTSD and severe asthma, included *ORMDL3* a gene known to be associated with asthma risk and STX8, which is involved in TrkA signaling. We also identified several pathways commonly regulated to both MDD and severe asthma. This study reveals gene and pathway regulation that potentially drives the comorbidity between severe asthma, PTSD, and MDD and may serve as foci for future research aimed at gaining a better understanding of both the relationship between asthma and PTSD, and the pathophysiology of the individual disorders.

Check for updates

OPEN ACCESS

Citation: Haas-Neill S, Dvorkin-Gheva A, Forsythe P (2022) Severe, but not moderate asthmatics share blood transcriptomic changes with post-traumatic stress disorder and depression. PLoS ONE 17(10): e0275864. https://doi.org/10.1371/journal.pone.0275864

Editor: Rohit Gaurav, University of Nebraska Medical Center, UNITED STATES

Received: May 4, 2022

Accepted: September 23, 2022

Published: October 7, 2022

Copyright: © 2022 Haas-Neill et al. This is an open access article distributed under the terms of the Creative Commons Attribution License, which permits unrestricted use, distribution, and reproduction in any medium, provided the original author and source are credited.

Data Availability Statement: NCBI's gene expression omnibus (GEO) database: https://www.ncbi.nlm.nih.gov/geo/.

Funding: This work was supported by funding from the Natural Sciences and Engineering Research Council of Canada (NSERC). PF is the AstraZeneca Canada Inc., Chair in Asthma and Obstructive Lung Disease.

Competing interests: The authors have declared that no competing interests exist.

Introduction

Asthma is a chronic inflammatory disease of the airways associated with recurrent episodes of wheezing, shortness of breath, chest tightness, and coughing. Generally, asthma is characterized by reversible constriction of the airways in response to allergen, but it can also be triggered by viral infection, physical activity, stress, or a negative mood [1]. Asthma affects 300 million

people worldwide and the World Health Organization has estimated that it is responsible for the loss of 15 million disability-adjusted life years (DALYs) annually [2, 3]. Asthma is also the most common chronic disease in children [4].

Epidemiological studies have shown significant association between asthma and mental health disorders, including anxiety, depression, panic attacks, and posttraumatic stress disorder (PTSD) [5–10].

MDD, more commonly referred to as 'depression' is a mental health disorder characterized by a low self-esteem, mood, and enjoyment of activities [11].

Studies have demonstrated consistent comorbidity between asthma and depression [7] and Youth with asthma are close to twice as likely to have anxiety and depressive disorders as those without asthma [8]. The co-occurrence of an anxiety or depressive disorder is associated with poor symptom control, impaired quality of life and increased health care utilization. While many studies have focused on psychosocial factors linking asthma and depression there is evidence that there may be shared pathophysiological factors between the diseases. For example, in a large-scale study in adults twins the association between depression and asthma remained significant after controlling for genetic and environmental factors [12]. However, the potential mechanisms and causality relating depression and asthma remain unclear [13–15].

PTSD is a mental health disorder that usually follows exposure to a traumatic event. The characteristic symptoms of PTSD include intrusive memories and nightmares, negative mood impaired cognition, avoidance behaviors, and changes to arousal behaviors such as increased irritability [16].

Clinical evidence supports a strong link between inflammatory conditions and PTSD with a particularly strong association between asthma and the prevalence and severity of PTSD [17, 18]. A twin study of Vietnam war veterans found that those with the top quartile of PTSD scores were 2-fold more likely to have asthma than those in the lower quartile [6]. This association was shown not to be predicted by familial or genetic factors, smoking, depression, or demographic factors [6]. Wisnivesky *et al*., (2021) [5] found that 19% of world trade center rescue and recovery workers with asthma also had PTSD, 10 times the prevalence in the general population. PTSD is also one of the greatest risk factors for decreased quality-of-life related to asthma [17, 18] and these poorer asthma outcomes do not appear to be due to differences in key asthma self-management behaviors [18]. Conversely, individuals with asthma prior to PTSD have been demonstrated to develop more aggravated asthma symptoms after the development of PTSD, while non-asthmatic subjects who develop PTSD have increased risk of adult onset asthma, suggesting a bidirectional relationship between these disorders [17].

An attempt by Jiang *et al*., (2014) [7] to identify a mechanism behind the comorbidity of asthma and MDD suggested immune factors may underlie both disorders. The investigation of 38 depression studies found that monocyte-derived, and other inflammatory cytokines (IL-1, IL-4, IL-6, and TNF) were significantly overexpressed in individuals with depression, while T cell derived cytokines (IL-10, and INF-γ) were uncorrelated with depression. Data comparing CD4+ T-cell expression in asthmatics with and without depression has also shown that 156 of 1448 total identified genes were differentially expressed in the depressed asthmatics group [19], suggesting that in circulating T-cells there is a unique transcriptomic profile for comorbid asthma and depression.

Genome-wide association studies (GWAS) have identified some shared genetic traits between those with asthma and MDD [20, 21]. In a cross-trait meta-analysis, Zhu *et al*., (2019) [20] identified 10 genomic loci shared between asthma and MDD and mendelian randomization identified a significant causal effect of MDD on asthma. The cross-trait meta-analysis performed by Cao *et al*., (2021) [21] identified 18 loci jointly associated between MDD and atopic diseases (asthma, eczema, and hay fever). Through Mendelian randomization analysis the

investigators found that MDD confers a stronger causal effect on those atopic diseases than they confer on MDD.

Similarly, in a meta-analysis by Nievergelt *et al*., (2019) [22], a pairwise genetic correlation demonstrated a high association between PTSD and asthma. Chronic stress, maternal stress, and more fundamentally, oxidative stress are also associated with severe asthma and increased asthma exacerbations [23–26]. Yan *et al*., 2021 [24] identified 12 genes methylated in individuals with exposure to chronic stress and violence, that were then shown to be associated with atopic childhood asthma. Although these studies were not looking at PTSD specifically, it is likely that genes associated with violence and chronic stress exposure would have close ties to those associated with PTSD.

Here, we downloaded 5 publicly available datasets from GEO, each of which compare one of PTSD, MDD, or asthma (a very large dataset which we split randomly into 2 datasets) blood transcription to that of healthy subjects. One dataset of each disease was used to explore genes and gene sets commonly shared between diseased subjects, and the other of each disease dataset was used to validate the genes and sets identified. Prior to conducting the investigation, we were interested in transcription specifically, as it facilitates functional change in the body and therefore we decided to compare the data to the hallmark, and C2 gene sets, which characterize canonical and curated changes in the body. Additionally, we hypothesized that as Jiang *et al*., (2014) [7] found immune factors involved in comorbidity, immune transcriptional changes commonly differentiated in whole blood would delineate the source of comorbidity. Immune factors have also been found partially responsible for cross talk between gut and brain in psycho-active probiotic treated mice exhibiting mood disorder-like symptoms [27–29]. For these reasons, we also compared these datasets to the C7 - immune signature gene set.

With a deeper understanding of the established comorbidity between mental health disorders and asthma, may come tangible knowledge on how to combat the root cause of these diseases and an expectation for how treatment of one disorder might affect another. Therefore, the goal of this study was to expand on genome-wide association studies by using publicly available data to characterize transcriptomic similarities between these disorders through analysis of genes and gene sets commonly differentially expressed between those suffering from the diseases and healthy subjects.

Results

Exploration of commonly differentially expressed genes

The 3 exploration datasets first underwent hierarchical clustering analysis, but there were no distinct clusters formed pertaining to diseased vs healthy subjects or along the lines of any other collected meta data. Principal component analysis was then used to check that no known variables could account for major differences that may arise during DE and GSEA analysis (Fig 1). There was no apparent grouping along PC1 or PC2 for any of the datasets, including for diseased vs healthy subjects (Fig 1). For the PTSD exploration cohort, 40.7% of the variance was accounted for by PC1, and 10.6% by PC2; for MDD, 16.8% of the variance was accounted for by PC1, and 6.2% by PC2; and for asthma, 22.7% of the variance was explained by PC1, and 10.0% by PC2.

Differential expression analysis of each disease to control subjects from their respective datasets reveals significant differences in both genes being up- and downregulated in all diseases (Fig 2). The analysis identified 8,321, 208, 1,736, and 373 genes significantly upregulated (adjusted p-value < 0.05; FC ≥ 1.5) in PTSD, MDD, severe asthma, and moderate asthma respectively, as compared to the corresponding controls. 7,062, 294, 2,735, and 901 genes were

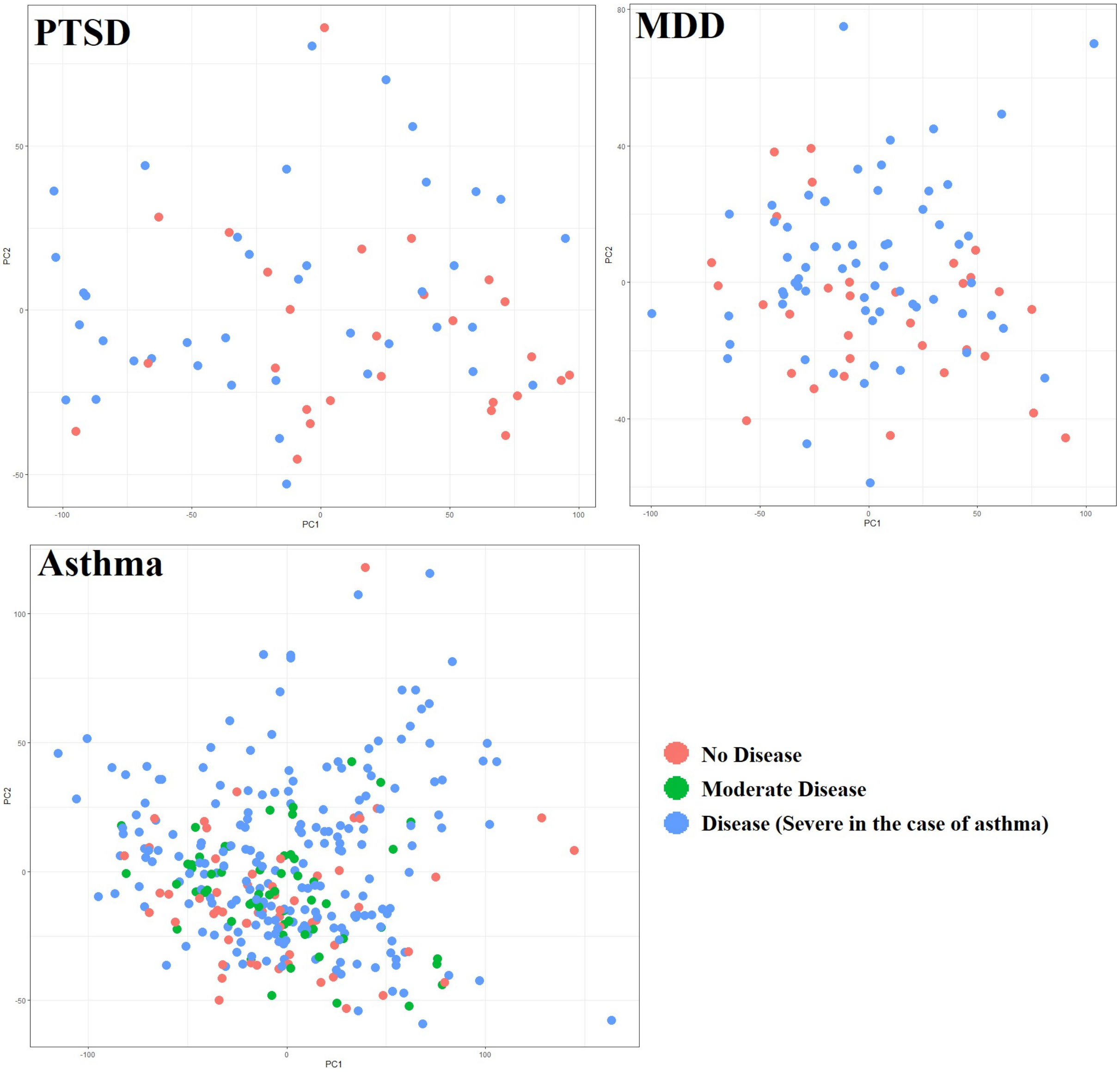

Fig 1. Principal component analysis. (PCA) showing PC1 and PC2 in each of the 3 disease exploration datasets.

https://doi.org/10.1371/journal.pone.0275864.g001

found to be significantly downregulated (adjusted p-value < 0.05; FC $\leq$ -1.5) in the same comparisons respectively.

Significantly regulated (adjusted P-value < 0.05, $|FC| \geq 1.5$) genes were compared between the exploration datasets for each disease. Genes found commonly to be regulated in the same direction in patients relatively to the healthy controls for multiple diseases were plotted in (Fig 3).

Exploration of commonly regulated gene sets

To detect the biological effect of more nuanced changes in all disease groups, Gene Set Enrichment Analysis (GSEA) was performed. GSEA compared expression of selected lists of genes (here termed "gene sets") between diseased and healthy subjects in each dataset (Fig 4). Gene sets from the Hallmark, C2, and C7 collections were compared against. Hallmark gene sets are

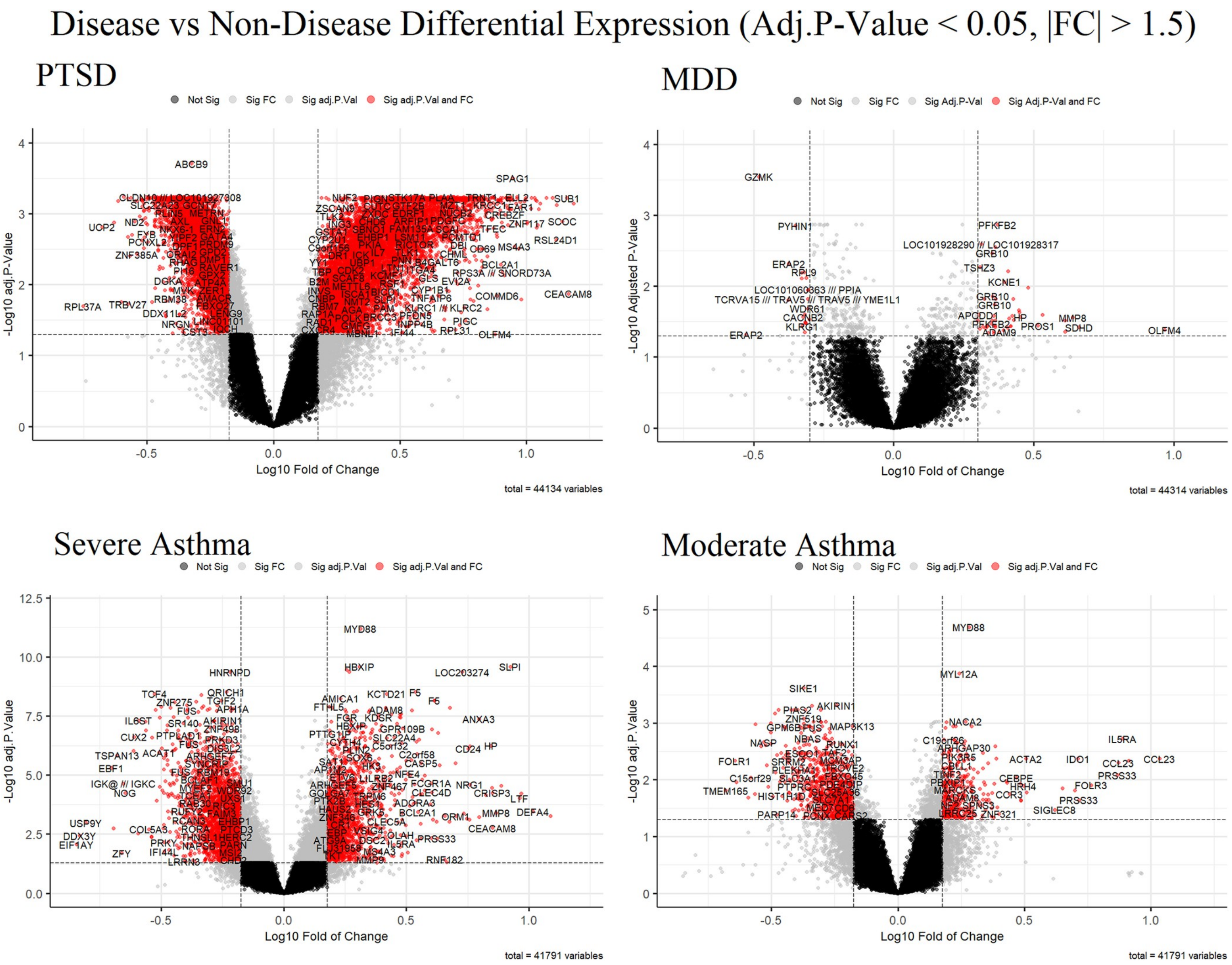

Fig 2. mRNA from the blood of subjects with a disease (PTSD, MDD, severe asthma, and moderate asthma) were compared to blood mRNA from non-diseased subjects for each exploration cohort dataset. The vertical threshold denotes genes or transcripts that are statistically significant (adjusted p-value < 0.05) while the horizontal threshold denotes genes or transcripts with an absolute fold change greater than 1.5. Genes or transcripts that meet none of these criteria are black, one of these criteria are grey, and both are red. The red genes, found to be significant, are also shown next to their symbols.

https://doi.org/10.1371/journal.pone.0275864.g002

sets of genes that comprise 50 of the best studied signaling pathways in the body. The C2 gene sets, or curated gene sets, in addition to the well understood and mapped 'KEGG pathways', include other sets of genes found previously to be differentially expressed in literature. C7 gene sets are immune signature gene sets found previously to be differentially expressed in literature.

No Hallmark gene sets were enriched in the same direction between all 4 datasets. 3 C2 gene sets were found to be upregulated in all 4 groups: REN_ALVEOLAR_RHABDOMYOSARCOMA_DN, JISON_SICKLE_CELL_DISEASE_UP, TAKEDA_TARGETS_OF_NUP98_HOXA9_FUSION_8D_DN, and REACTOME_NEUTROPHIL_DEGRANULATION. No C2 gene sets were commonly downregulated in all 4 groups. 65 C7 gene sets were commonly upregulated in all 4 groups, but nothing was commonly downregulated in all 4 of those groups.

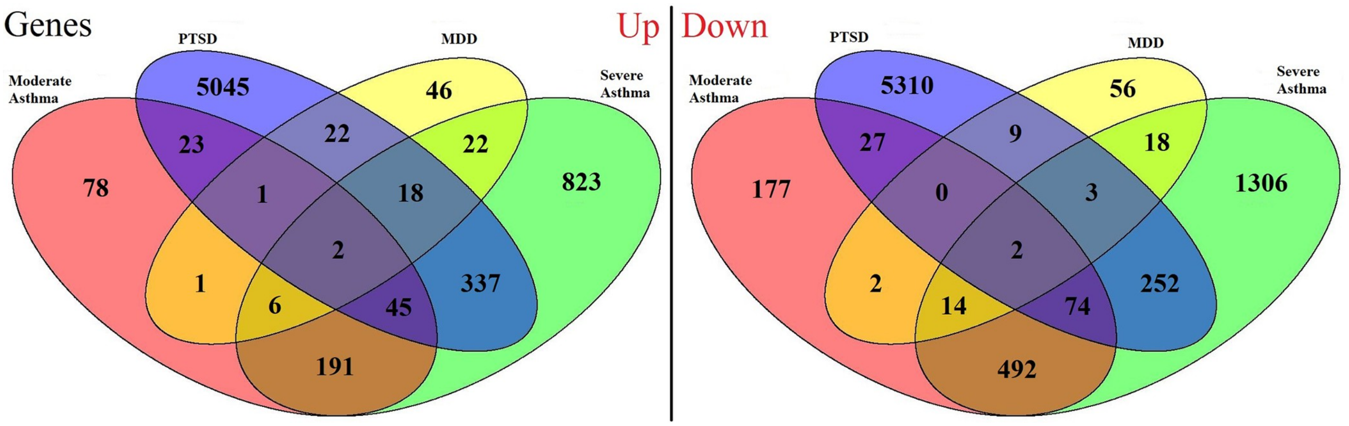

Fig 3. The number of genes differentially expressed from healthy subjects in the same direction between different diseases. The numbers within the different overlaps of the venn diagram are the number of genes significantly (adjusted P-value < 0.05) differentially expressed in both exploration datasets. For example, in the left 'up' panel, there are 22 genes in the PTSD and MDD exploration sets that are similarly significantly overexpressed, and in the right 'down' panel, there are 2 genes commonly underexpressed in all disease exploration datasets compared to their respective healthy controls.

https://doi.org/10.1371/journal.pone.0275864.g003

Validation of differentially expressed genes in independent transcriptomic datasets

To challenge these findings, the 'validation' datasets for each of: MDD, PTSD, severe asthma, and moderate asthma underwent DE analysis with limma. No genes were found to be significantly regulated (adjusted P-value < 0.05) in the same directions for all 4 sets as no individual genes were significantly differentially expressed in the MDD validation dataset. 2 genes were validated as upregulated in PTSD and severe asthma: *STX8* (Adjusted p-values in PTSD exploration, PTSD validation, severe asthma exploration, severe asthma validation were: 1.6E-3, 1.8E-2, 2.6E-3, 3.7E-4) and *ARHGAP24* (1.4E-2, 1.6E-2, 3.9E-2, 3.2E-2). Commonly downregulated to PTSD and severe asthma were *ORMDL3* (2.2E-2, 1.9E-3, 2.7E-3, 3.9E-3), *PTP4A3* (2.6E-3, 2.3E-2, 4.5E-3, 5.2E-3), *SHISA4* (1.1E-2, 4.4E-2, 9.8E-3, 6.2E-3), and *TPPP3* (2.2E-2, 1.1E-2, 3.1E-3, 2.7E-2). No differentially expressed genes were validated between PTSD and moderate asthma in either direction, however. 582 genes were validated as significantly downregulated between moderate and severe asthma while no upregulated genes could be validated.

Validation of regulated pathways in independent transcriptomic datasets

The same datasets used to validate differentially expressed genes were used to validate gene sets and pathways identified as being commonly regulated in either direction in the exploration datasets. Interestingly, despite no genes being significantly differentially expressed in MDD patients vs healthy controls in the validation dataset, there were pathways identified as being significantly altered in severe asthma patients as compared to their corresponding controls (Table 1).

As may be expected, many pathways were found to be commonly modified between moderate and severe asthma when comparing against the C2 and C7 gene sets and although they are not the focus of this study on comorbidity, can be found listed in supplementary information (S1 Table in S1 File). Barcode plots showing a more detailed cross-section of gene expression from the sets in Table 1 can be found in supplementary information (S1-S6 Figs in S1 File).

Finally, we pooled all genes from each significantly differentially expressed set common to MDD and severe asthma (Table 1) and performed a STRING cluster analysis for proteins to determine if any other functional networks emerged. Two networks were examined, grouping

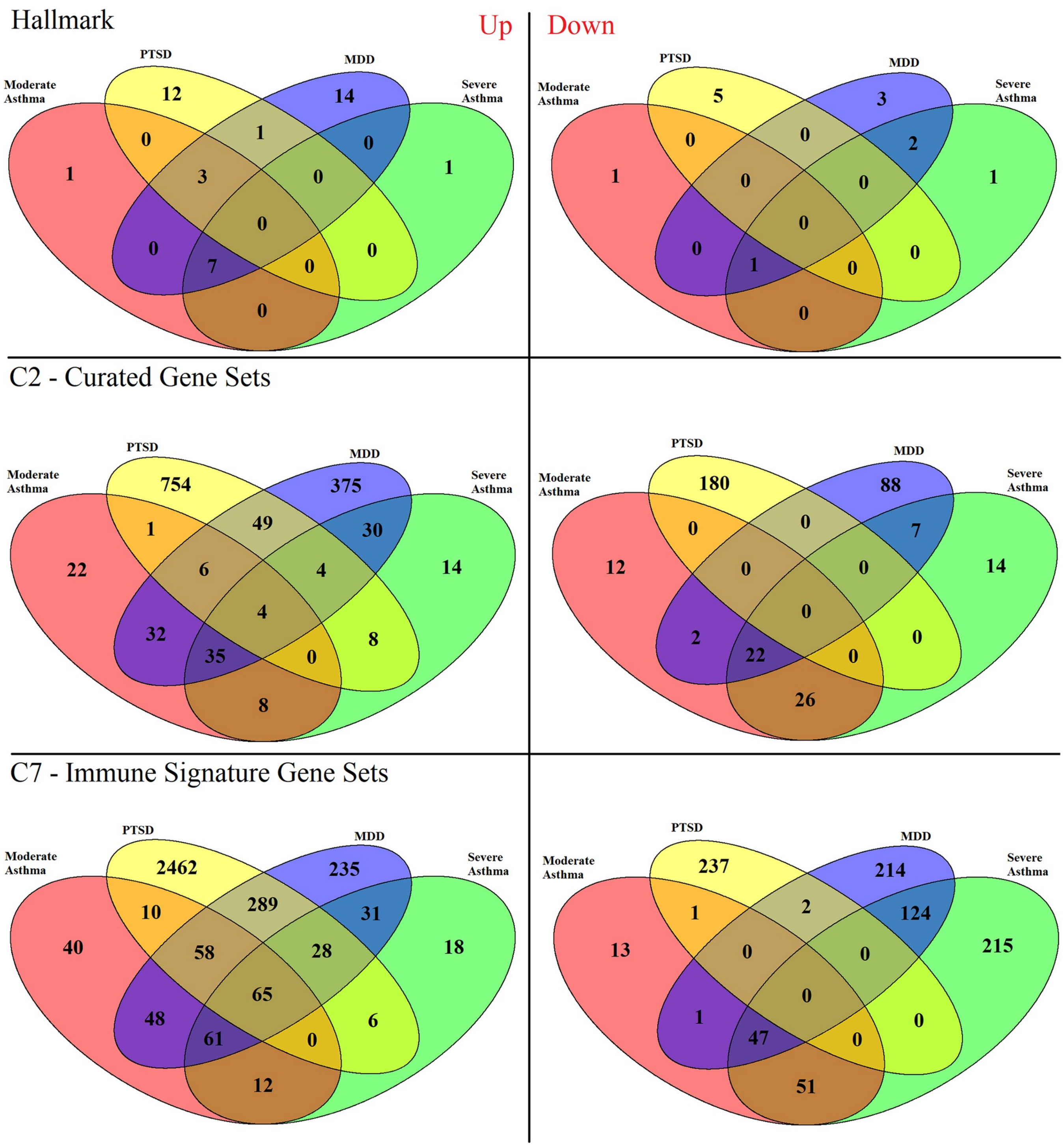

Fig 4. Gene set enrichment analysis. (GSEA) showing significantly (adjusted P-value < 0.05) modified pathways between all 4 exploration datasets. Labels identify the gene sets being compared to and in which direction (left = upregulated, right = downregulated).

https://doi.org/10.1371/journal.pone.0275864.g004

genes enriched in both MDD and severe asthma compared to healthy subjects, as well as genes enriched in healthy subjects compared to MDD and severe asthma (Fig 5). Among many other associations, STRING analysis found that proteins encoded by the disease-enriched genes of

Table 1. Directionally validated pathway comparisons in the Hallmark, C2, and C7 collections.

Direction	Comparison	Enriched Gene Set	Adjusted P-Values
Up	MDD and Severe Asthma C7	GSE4748_CYANOBACTERIUM_LPSLIKE_VS_LPS_AND_CYANOBACTERIUM_LPSLIKE_STIM_DC_3H_DN	MDD1 - 1.58e-11, MDD2 - 1.30e-2, S. Asthma1 - 5.49e-14, S.Asthma2 - 3.42e-12
		GSE34205_HEALTHY_VS_RSV_INF_INFANT_PBMC_DN	MDD1 - 7.69e-12, MDD2 - 1.16e-2, S. Asthma1 - 1.79e-10, S.Asthma2 - 6.29e-10
Down	MDD and Severe Asthma C7	GSE22886_NAIVE_BCELL_VS_NEUTROPHIL_UP	MDD1 - 1.30e-10, MDD2 - 2.61e-3, S. Asthma1 - 4.41e-7, S.Asthma2 - 2.27e-9
		GSE34205_HEALTHY_VS_FLU_INF_INFANT_PBMC_UP	MDD1 - 4.69e-3, MDD2 - 2.26e-2, S. Asthma1 - 4.47e-3, S.Asthma2 - 9.99e-6
		GSE22886_NEUTROPHIL_VS_MONOCYTE_DN	MDD1 - 3.37e-2, MDD2 - 7.46e-8, S. Asthma1 - 3.74e-2, S.Asthma2 - 3.40e-3
Down	MDD and Severe Asthma C2	JISON_SICKLE_CELL_DISEASE_DN	MDD1 - 1.37e-8, MDD2 - 4.42e-2, S. Asthma1 - 4.44e-3, S.Asthma2 - 9.13e-5

Directionally validated pathway comparisons in the Hallmark, C2, and C7 collections, following GSEA excluding comparison between severe and moderate asthma.

https://doi.org/10.1371/journal.pone.0275864.t001

the "GSE4748_CYANOBACTERIUM_LPSLIKE_VS_LPS_AND_CYANOBACTERIUM_LPSLIKE_STIM_DC_3H_DN," and "GSE34205_HEALTHY_VS_RSV_INF_INFANT_PBMC_DN" gene sets have been previously identified in literature in various roles - including modulation of immune function, cancer involvement, and more (Fig 5A). The complete list of functional annotations can be found in S1 and S2 Data.

Likewise, proteins encoded by the healthy subject-enriched genes of the "GSE22886_NAIVE_BCELL_VS_NEUTROPHIL_UP," "GSE34205_HEALTHY_VS_FLU_INF_INFANT_PBMC_UP," "GSE22886_NEUTROPHIL_VS_MONOCYTE_DN," and "JISON_SICKLE_CELL_DISEASE_DN" gene sets have been shown involved in several functional enrichments including several facets of ribosome regulation, and MHC class II activity. The complete list of functional annotations can be found in S1 and S2 Data.

Discussion

While it is widely accepted that psychosocial factors affect asthma pathobiology in children and adults, there is little understanding of potential common biological pathways underlying comorbidity between asthma and mental health disorders. Previous reports based on GWAS studies were focusing on determination of shared genetic traits between Asthma, MDD and PTSD or on circulating levels of specific inflammatory cytokines to explore potential shared pathophysiology of these disorders. In an attempt to provide further insight into the comorbidity of these conditions and to identify target pathways for further investigation, we utilized publicly available data to assess similarities between asthma, MDD and PTSD at the transcriptomic level.

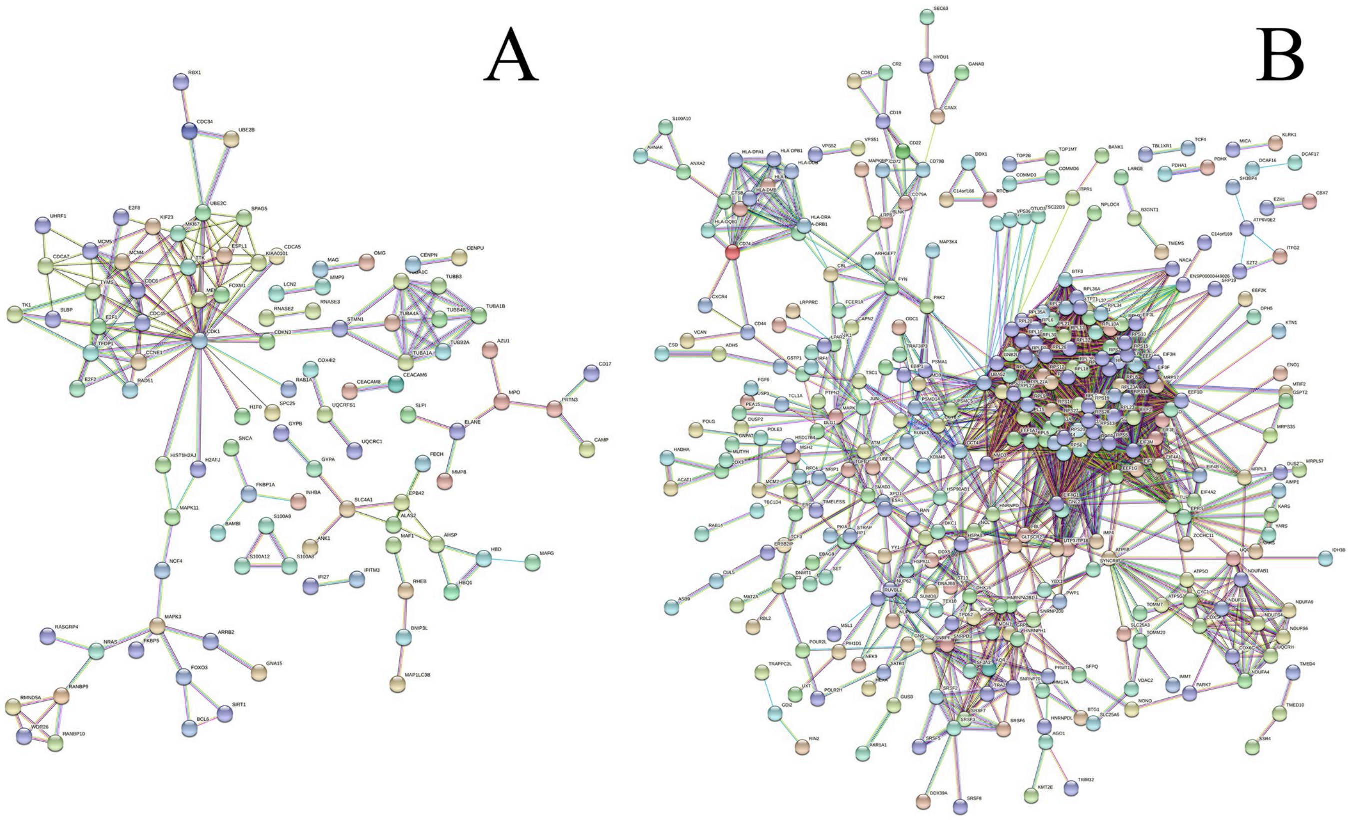

Fig 5. STRING analysis for proteins coded by genes. Found A) enriched in the peripheral whole blood of both MDD and severe asthma patients compared to healthy subjects, and B), enriched in healthy subjects compared to both MDD and severe asthma patients. Only high confidence interactions between proteins are shown, and proteins that are not involved in a high confidence interaction do not appear.

https://doi.org/10.1371/journal.pone.0275864.g005

As expected, asthma, MDD and PTSD were associated with many differentially expressed genes and gene sets, and, in comparing exploration cohorts, a number of these genes and gene sets were significantly regulated in the same direction in all diseases. Upon validation, commonalities in transcriptomic changes were restricted to comparisons between severe asthma and MDD or PTSD.

In keeping with literature indicating a close association with regards to comorbidity and reciprocal enhancement of symptom severity [17, 18], our cross-disease comparisons found the greatest transcriptomic level similarities between severe asthma and PTSD.

With regard to commonly differentially expressed genes we found *ORMDL3* to be downregulated in the blood of both PTSD and severe asthma subjects. *ORMDL3* codes for a protein called "ORMDL sphingolipid biosynthesis regulator 3" which resides in the endoplasmic reticulum and is a regulator of sphingolipid synthesis [30]. ORMDL3 requires precise expression to function correctly - under normal conditions it inhibits the rate limiting enzyme of sphingolipid biosynthesis, serine palmitoyl transferase (SPT) [30]. Downstream of uninhibited SPT activity, ceramide - the central sphingolipid metabolite - is produced and transported to the golgi [31]. Therefore, a knockdown of ORMDL3 can result in an abundance of ceramide [32]. When slightly overexpressed, ORMDL3 leads to a dearth of ceramide, however, when highly overexpressed, ORMDL3 increases ceramide biosynthesis through the alternate, recycling/salvage pathway [33, 34].

Numerous GWAS have identified ORMDL3 as a potential susceptibility gene for asthma and polymorphisms controlling ORMDL3 expression have been associated with both asthma occurrence and exacerbation [34–41].

However, the mechanistic contribution of ORMDL3 to the pathogenesis of asthma remains unclear and experimental evidence suggests the relationship between ORMDL3 and asthma is complex. Studies in animal models of allergic airway inflammation have indicated that overexpression of ORMDL3 leads to increased ceramide levels and the accompanying ER stress leads to characteristic features of asthma including increased mucus production, an exacerbated inflammatory response, and airway hyperresponsiveness. Correspondingly, downregulation of ORMDL3 expression, and decreased ceramide levels, were demonstrated to significantly ameliorate asthmatic symptoms in a mouse model [33, 42–46]. Furthermore, the expression of ORMDL3 in eosinophils seems to play a role in recruitment, attachment and activation of eosinophils in asthma [47]. However, seemingly conflicting evidence suggests that decreased expression of ORMDL3 can also promote asthma symptoms. Selective knockdown of ORMDL3 in lung epithelial cells leads to airway hyperresponsiveness [48], while downregulation of ORMDL3 in mast cells, cells key to asthma pathogenesis, enhances antigen mediated expression of proinflammatory cytokines and production of prostaglandin D2 and promotes mast cell driven inflammation in vivo [49].

While, to our knowledge there have been no studies associating ORMDL3 and PTSD, ceramide is a precursor for complex sphingolipids that are highly abundant in neural cellular membranes and are regulators of brain homeostasis [50]. Ceramide has also been shown to promote stress-induced depression-like behavior in mice, and intervention with drugs that reduce hippocampal ceramide (amitriptyline and fluoxetine) rescued those behaviours [51, 52].

Upregulated in the blood of severe asthma and PTSD subjects were mRNA encoding Syntaxin 8 (STX8), and Rho GTPase Activating Protein 24 (ARHGAP24). STX8 is a t-SNARE protein (target soluble N-ethylmaleimide-sensitive factor attachment protein receptor) involved in diverse vesicle docking and membrane fusion events. STX8 has been demonstrated to regulate the function of receptors and ion channels, including TrkA and CFTR. The TrkA receptor is transported from the golgi to the plasma membrane by STX8, a process which with nerve growth factor (NGF) stimulation promotes downstream TrkA signaling [53]. Interestingly, higher levels of TrkA expression have been identified in patients with allergic asthma [54], and although its role in asthma has not been fully elucidated, there are several proposed mechanisms by which neurotrophin signaling exacerbates asthma [55]. Some evidence suggests neurotrophin signaling may modulate airway hyperactivity and bronchoconstrictor release, enhancement of airway contractility, as well as airway remodeling [55–57]. TrkA has also been previously implicated in PTSD, as NGF signaling via TrkA alleviated stress induced PTSD-like symptoms in mice [58]. In contrast to enhancing TrkA signaling, STX8 also interacts with CFTR to inhibit function and trafficking to the cell surface [59]. CFTR is largely studied in relation to cystic fibrosis, however, impaired function of this ion channel has been associated with more severe or difficult to treat asthma [60–62]. While to our knowledge there has been no suggested relationship between CFTR and PTSD, the ion channel is expressed throughout the central nervous system [63].

ARHGAP24 converts the Rac-type GTPase into its inactive GDP-bound state which, downstream of Rho, suppresses actin remodelling [64]. Increased activation of RhoA/Rho-kinase is associated with airway hyper-responsiveness and smooth muscle contraction in asthma [65]. Cerebral RhoA activation is known to enhance fear memory which may have implications for PTSD [66]. So, in both asthma and PTSD, increased Rho activity is associated with increased pathology. It is curious then, that we find an inhibitor of its downstream activity differentially

overexpressed in the blood of the diseased subjects. The reason for this would have to be elucidated by further research.

Other genes commonly downregulated in severe asthma and PTSD were Protein Tyrosine Phosphatase 4A3 (PTP4A3), known for its role in stimulating progression from G1 to S phase in mitosis [67]; Shisa Family Member 4 (SHISA4), a transmembrane scaffold/adaptor protein [68]; and Tubulin Polymerization Promoting Protein Family Member 3 (TPPP3), a regulator of microtubule dynamics [69]. To our knowledge, none of these proteins have previously been associated with asthma or PTSD and their identification here may warrant further investigation.

Neither ORMDL3, STX8, nor ARHGAP24 are discussed by Bigler *et al*., (2017) [70] in relation to the asthma datasets; nor are they identified in the PTSD dataset by Rusch *et al*., (2019) [71]. ARHGAP24 is discussed briefly in regards to PTSD in the validation data set, (Kuan *et al*., 2017) [72] as being a member of the PTSD-associated actin cytoskeleton pathway.

One of the gene sets "GSE34205_HEALTHY_VS_RSV_INF_INFANT_PBMC_DN" refers to a list of genes found to be more highly expressed in peripheral blood mononuclear cells (PBMC) of infants with RSV (Respiratory syncytial virus) bronchiolitis [69] when compared to those of healthy subjects. We also found that "GSE34205_HEALTHY_VS_FLU_INF_INFANT_PBMC_UP," a list of genes with decreased in expression infants with acute influenza compared to PBMCs of healthy subjects, was downregulated in both MDD and severe asthma [73]. These 2 congruent pieces of evidence suggest that the immune signature to respiratory infection in infants is similar to the immune signature of both asthma and MDD whole blood. In human airway epithelial cells Ioannidis *et al*., (2012) [73] found that comparing both influenza and RSV treatment to control exhibited DE reminiscent of a type I interferon immune signature and genes downstream of IFN-α/β were expressed abundantly in infected cells. Type I interferon signaling is known to be a contributing factor in some cases of both depression and asthma [74–77].

Two additional gene sets we found downregulated in both MDD and severe asthma: "GSE22886_NAIVE_BCELL_VS_NEUTROPHIL_UP," and "GSE22886_NEUTROPHIL_VS_MONOCYTE_DN" were both compiled by Abbas *et al*., (2005) [78] to identify patterns in immune cell-specific expression in order to identify states of activation. The gene sets we identified as being underexpressed in MDD and severe asthma can be congruently explained by a reduction of neutrophil specific gene expression, or by an increase in naive B-cell and monocyte specific gene expression. The latter is perhaps more likely as neutrophils have been demonstrated to be activated in patients with MDD and asthma [79, 80]. Furthermore, B cell homeostasis is altered in individuals with MDD and B cells play a crucial role in regulating the hyperactivity of airways in asthma [81–84]. Likewise, there is generally increased activity and larger numbers of monocytes in MDD and asthma compared to healthy subjects [85–88]. This highlights the possibility of enhanced B cell and monocyte activity playing a key role in comorbid asthma and MDD.

JISON_SICKLE_CELL_DISEASE_DN, found downregulated in the blood for both MDD and severe asthma, are genes previously found to be downregulated in peripheral blood mononuclear cells (PBMCs) in sickle-cell disease patients compared with non-diseased counterparts. Asthma is common in children with sickle cell disease and this comorbidity is becoming increasingly well documented [89]. In sickle cell, nitric oxide consumption mediated by plasma hemoglobin, ischemia-reperfusion injury, and the generation of free radicals activate an inflammatory stress response [89]. Jison *et al*., (2004) [90], who discovered the gene set, found many of the genes differentially expressed within PBMCs were linked to inflammatory stress as well. To find these same genes underexpressed in two comorbid conditions suggests

that the inflammatory stress response itself could be a driver behind comorbidity for sickle cell disease, MDD, and severe asthma.

The STRING analysis for proteins translated from the individual genes in the gene sets commonly regulated between MDD and severe asthma show that the genes upregulated in each of these diseases have several functional associations. By combining curated gene sets enriched in both diseases we gave the string analysis a more complete picture of all the systems that may be modified downstream of these blood transcriptional changes. In addition to basic biological processes, cellular compartments, molecular functions, and pathways, several smaller literature-backed gene sets were found in common. Examining the top 5 in descending order of strength our genes enriched in MDD and severe asthma, we observed matches to biomarkers for severe influenza infection (Adj. P-value = 2.2E-5) [91], genes associated with arthritis (Adj. P-value = 1.5E-3) [92], respiratory distress syndrome phenotypes (Adj. P-value = 1.2E-2) [93], lung epithelial function in sepsis (Adj. P-value = 5.6E-6) [94], and myocardial infarction and neutrophil degranulation (Adj. P-value = 2.6E-4) [95]. Looking at the top 5 for genes enriched in healthy subjects compared to MDD and severe asthma we identified many matches associated with ribosomal regulation, and to a lesser extent immune function and anemia (Adj. P-values = 3.1E-9, 2.0E-4, 2.0E-4, 1.3E-3, 6.5E-3) [96–100]. This could suggest that there is less ribosomal regulation in MDD and severe asthma, and further suggests that immune involvement could drive the relationship between these disorders.

Despite MDD being a major comorbidity in PTSD, and 440 immune signature gene sets commonly upregulated between the exploration datasets, no genes or gene sets were validated in this study when comparing MDD and PTSD. However, the neurobiology of the link between PTSD and MDD is unclear and it is entirely feasible that similarities in gene expression between the disorders is restricted to the CNS and are undetectable in the blood.

It is notable that there were no validated genes or gene sets in common between mild/moderate asthma and either of the mental health disorders. This finding is consistent with the phenomenon that mental health disorders such as PTSD and MDD are correlated with more severe disease outcomes [101]. It may be that activation of specific genes or pathways that are involved in MDD or PTSD are also factors that contribute to the development of more severe asthma. In this regard, there is evidence to suggest that antidepressant treatment improved asthma symptoms in severe but not mild asthmatics with co-morbid depression.

Overall, with six parallel DGE analyses and GSEA on whole blood gene expression, we identified genes and gene set expression that potentially links severe asthma to both PTSD and MDD. The gene sets commonly regulated between asthma and MDD, support previously suggested links between inflammation related immune factors and the two disorders [7]. Epidemiological evidence indicating that PTSD has a stronger association with asthma than other chronic inflammatory diseases [102, 103] suggests that the relationship is driven by more than common immune factors. Here we identify 6 genes (2 upregulated in disease and 4 downregulated) being differentially expressed in both PTSD and asthma. Of particular note, our results identify mechanisms involving ceramide biosynthesis and SNARE regulated signaling pathways as potential targets for future research aimed at understanding both the relationship between PTSD and asthma and the pathophysiology of the individual disorders.

Methods

Obtaining and preprocessing datasets

Data were downloaded from the Gene Expression Omnibus (GEO) repository and preprocessed using the methods described by the respective authors associated with each dataset (Table 2). Specific blood RNA datasets were chosen over others on GEO due to there being

Table 2. List of datasets used in this paper.

GSE #	Platform (GPL)	Source and Species	Normalization Method	Purpose	Associated Publication	# Samples	# Genes / Variants
GSE81761	GPL570 (Array)	Human whole blood mRNA	RMA*	PTSD Exploration	Rusch *et al.*, 2019	27 - PTSD, 39 - No PTSD	44,134
GSE97356	GPL11154 (RNAseq)	Human whole blood mRNA	TMM	PTSD Validation	Kuan *et al.*, 2017	82 - PTSD, 201 - No PTSD	15,112
GSE98793	GPL570 (Array)	Human whole blood RNA	RMA	MDD Exploration	Leday *et al.*, 2018	64 - MDD, 32 - No MDD	44,134
GSE19738	GPL6848 (Array)	Human whole blood RNA	Quantile	MDD Validation	Spijker *et al.*, 2010	33 - MDD, 34 - No MDD	12,816
GSE69683	GPL13158 (Array)	Human whole blood RNA	RMA	Asthma Exploration and Validation	Bigler *et al.*, 2017	After Split:<br>Exploration: 58 - Healthy, 58 - Moderate, 216 - Severe.<br>Validation: 28 - Healthy, 20 - Moderate, 128 - Severe	41,791

List of datasets used in this paper with a description of data type, preprocessing, number of genes and gene variants remaining in the dataset following preprocessing, and associated publications.

* Robust multichip average (RMA) normalization.

https://doi.org/10.1371/journal.pone.0275864.t002

among the few datasets on GEO that met the specific criteria of whole blood (rather than PBMCs, or biopsy), the specific diseases in question, and focused on mRNA (rather than total RNA or miRNA). Any remaining appropriate datasets on GEO were on different platforms. We decided against pooling these datasets since attempts to correct for technical variation forces data modification that can confound and obscure the true biological variation of interest, and increase the likelihood of generating erroneous results. Therefore, we preferred to select the largest available datasets that did not require pooling for a classic exploration and validation analysis.

Rusch *et al*., (2019) [71] (preprocessed and raw data available at: https://www.ncbi.nlm.nih.gov/geo/query/acc.cgi?acc=GSE81761) measured blood mRNA military service members, with and without PTSD. Only samples from the first time-point collection, rather than the follow up collection, were selected for analysis. Other information collected on the subjects included sex (63 male, 3 female), age (22-49), and race. Kuan *et al*., (2017) [72] (preprocessed and raw data available at: https://www.ncbi.nlm.nih.gov/geo/query/acc.cgi?acc=GSE97356) measured blood mRNA in World Trade Center responders with PTSD currently, never, and in the past. Samples collected from subjects who never had PTSD or had PTSD at the time of the collection were selected for further analysis. No other sample information was supplied with the dataset. Leday *et al*., (2018) [104] (preprocessed and raw data available at: https://www.ncbi.nlm.nih.gov/geo/query/acc.cgi?acc=GSE98793) pooled human blood mRNA data from two depression studies: the "Janssen–Brain Resource Company "study, and the "GlaxoSmithKline–High-Throughput Disease-specific target Identification Program" study into subjects with MDD, and without. Batch 1 and batch 2 were originally found to generate distinct groups in principal component analysis (PCA), and were batch corrected with the 'removeBatchEffect' function in limma package (Ritchie *et al*., 2015 [105]) in R. This dataset contained additional information, such as including gender (144 female, 48 male), age (31-72), and anxiety status (128 no, 64 yes). Spijker *et al*., (2010) [106] (preprocessed and raw data available at: https://www.ncbi.nlm.nih.gov/geo/query/acc.cgi?acc=GSE19738) collected blood from subjects with and without MDD prior to and following stimulation with lipopolysaccharide (LPS), data which we excluded. Additional information in the dataset was age (21-63), gender (41

female, 26 male), and smoking status (20 non-smoking, 18 quit smoking, 29 smoking). The Unbiased Biomarkers for the Prediction of Respiratory Disease Outcomes (U-BIOPRED) study dataset (Bigler *et al*., 2017) [70] (preprocessed and raw data available at: https://www.ncbi.nlm.nih.gov/geo/query/acc.cgi?acc=GSE69683) measured blood mRNA in subjects with moderate (lung function tests are 60-80% of expected value), severe (lung function tests are <60% of expected value), and no asthma. The dataset also contained information on gender of the patients (275 female, 223 male), smoking or non-smoking (410 non-smoking, 88 smoking). We randomly divided this dataset into an exploration and a validation cohort at a 2:1 ratio. Low expressed genes were filtered out prior to trimmed mean of M-values (TMM) normalization of the RNAseq dataset as it is more sensitive due to its single nucleotide resolution [107, 108]. This was performed using the edgeR packages 'filterByExpr' function [109]. Data on race was only available in the Rusch *et al*., (2019), and Bigler *et al*., (2017) datasets and both studies had predominantly white caucasian participants (66% and 90%, respectively).

Seeing as not all datasets contained the same background information on their respective subjects, and because the purpose of this study was to detect commonalities between comorbid diseases that may exist robustly in a particular disease regardless of other variables, demographic information such as age, race, gender, and smoking status was not taken into consideration.

Principal component analysis (PCA) was done in base R and visualized using ggplot2 [110]. Venn diagrams were generated using the VennDiagram R package [111].

Differential gene expression

Each dataset, including the split asthma datasets for both severe and moderate asthma, underwent differential gene expression analysis individually, comparing their disease to the respective control group (the non-disease group) from the same study. Analysis was performed using the limma package with multiple hypothesis correction and Benjamini-Hochberg FDR applied. Genes were considered to be differentially expressed with an adjusted p-value < 0.05 and $|FC| \geq 1.5$.

Gene set enrichment analysis. 3 MSigDB collections of gene sets (v7.4) were downloaded from the GSEA website (https://www.gsea-msigdb.org/gsea/msigdb/index.jsp): Hallmark - well-defined biological states or processes, C2 - curated gene sets from PubMed publications and online pathway databases (including KEGG), and C7 - immunologic signature gene sets representative of immune and cell states.

Fold change values generated by the differential expression analysis of diseased subjects vs healthy subjects were compared to each of the 3 collections via their entrez gene IDs using the Gage package in R [112]. Gage uses the differential expression output of all genes, not just those with significant fold change or p-value. Barcode plots were generated using barcodeplot () function (limma package). Volcano plots were generated using the R package 'Enhanced-Volcano' [113].

Supporting information

S1 File.
(DOCX)

S1 Data.
(TSV)

S2 Data.
(TSV)

Author Contributions

Conceptualization: Anna Dvorkin-Gheva, Paul Forsythe.

Formal analysis: Sandor Haas-Neill.

Investigation: Sandor Haas-Neill.

Methodology: Anna Dvorkin-Gheva.

Supervision: Paul Forsythe.

Writing – original draft: Sandor Haas-Neill, Paul Forsythe.

Writing – review & editing: Sandor Haas-Neill, Anna Dvorkin-Gheva, Paul Forsythe.

References

1. Janssens T, Ritz T. Perceived triggers of asthma: key to symptom perception and management. Clin Exp Allergy [Internet]. 2013 Sep; 43(9):1000–8. Available from: https://onlinelibrary.wiley.com/doi/10.1111/cea.12138 PMID: 23957335
2. Masoli M, Fabian D, Holt S, Beasley R. The global burden of asthma: executive summary of the GINA Dissemination Committee Report. Allergy [Internet]. 2004 May; 59(5):469–78. Available from: https://onlinelibrary.wiley.com/doi/10.1111/j.1398-9995.2004.00526.x PMID: 15080825
3. Vos T, Lim SS, Abbafati C, Abbas KM, Abbasi M, Abbasifard M, et al. Global burden of 369 diseases and injuries in 204 countries and territories, 1990–2019: a systematic analysis for the Global Burden of Disease Study 2019. Lancet [Internet]. 2020 Oct; 396(10258):1204–22. Available from: https://linkinghub.elsevier.com/retrieve/pii/S0140673620309259 https://doi.org/10.1016/S0140-6736(20)30925-9 PMID: 33069326
4. Centers for Disease Control and Prevention NC for HS. 2011-2012 National Survey of Children's Health Frequently Asked Questions [Internet]. 2013. Available from: https://www.cdc.gov/nchs/slaits/nsch.htm
5. Wisnivesky JP, Markowitz SB, James S, Stone K, Dickens B, Busse P, et al. Comorbid posttraumatic stress disorder and major depressive disorder are associated with asthma morbidity among World Trade Center workers. Ann Allergy, Asthma Immunol [Internet]. 2021 Mar; 126(3):278–83. Available from: https://linkinghub.elsevier.com/retrieve/pii/S1081120620311078 https://doi.org/10.1016/j.anai.2020.10.007 PMID: 33098982
6. Goodwin RD, Fischer ME, Goldberg J. A Twin Study of Post–Traumatic Stress Disorder Symptoms and Asthma. Am J Respir Crit Care Med [Internet]. 2007 Nov 15; 176(10):983–7. Available from: http://www.atsjournals.org/doi/abs/10.1164/rccm.200610-1467OC PMID: 17702964
7. Jiang M, Qin P, Yang X. Comorbidity between depression and asthma via immune-inflammatory pathways: A meta-analysis. J Affect Disord [Internet]. 2014 Sep; 166:22–9. Available from: https://linkinghub.elsevier.com/retrieve/pii/S0165032714002110 https://doi.org/10.1016/j.jad.2014.04.027 PMID: 25012406
8. Katon W, Lozano P, Russo J, McCauley E, Richardson L, Bush T. The Prevalence of DSM-IV Anxiety and Depressive Disorders in Youth with Asthma Compared with Controls. J Adolesc Heal [Internet]. 2007 Nov; 41(5):455–63. Available from: https://linkinghub.elsevier.com/retrieve/pii/S1054139X07002443 https://doi.org/10.1016/j.jadohealth.2007.05.023 PMID: 17950165
9. Caramanica K, Brackbill RM, Liao T, Stellman SD. Comorbidity of 9/11-Related PTSD and Depression in the World Trade Center Health Registry 10-11 Years Postdisaster. J Trauma Stress [Internet]. 2014 Dec; 27(6):680–8. Available from: https://onlinelibrary.wiley.com/doi/10.1002/jts.21972 PMID: 25470556
10. Rytwinski NK, Scur MD, Feeny NC, Youngstrom EA. The Co-Occurrence of Major Depressive Disorder Among Individuals With Posttraumatic Stress Disorder: A Meta-Analysis. J Trauma Stress [Internet]. 2013 Jun; 26(3):299–309. Available from: https://onlinelibrary.wiley.com/doi/10.1002/jts.21814 PMID: 23696449
11. Kennedy SH. Core symptoms of major depressive disorder: relevance to diagnosis and treatment. Dialogues Clin Neurosci [Internet]. 2008 Sep 30; 10(3):271–7. Available from: https://www.tandfonline.com/doi/full/10.31887/DCNS.2008.10.3/shkennedy PMID: 18979940
12. Tedner SG, Lundholm C, Olsson H, Almqvist C. Depression or anxiety in adult twins is associated with asthma diagnosis but not with offspring asthma. Clin Exp Allergy [Internet]. 2016 Jun; 46(6):803–12. Available from: https://onlinelibrary.wiley.com/doi/10.1111/cea.12714 PMID: 27228571

13. Hasler G, Gergen PJ, Kleinbaum DG, Ajdacic V, Gamma A, Eich D, et al. Asthma and Panic in Young Adults. Am J Respir Crit Care Med [Internet]. 2005 Jun; 171(11):1224–30. Available from: http://www.atsjournals.org/doi/abs/10.1164/rccm.200412-1669OC

14. To T, Ryckman K, Zhu J, Williams D, Feldman LY, Larsen K, et al. Mental Health Services Claims and Adult Onset Asthma in Ontario, Canada. J Allergy Clin Immunol Pract [Internet]. 2017 Sep; 5(5):1388–1393.e3. Available from: https://linkinghub.elsevier.com/retrieve/pii/S2213219817301137 PMID: 28396111

15. Vargas PA. Spreading the Word: Comorbidity of Asthma and Depression Is Not Just the Product of a Vulnerable Personality. J Allergy Clin Immunol Pract [Internet]. 2020 Jan; 8(1):208–9. Available from: https://linkinghub.elsevier.com/retrieve/pii/S2213219819306415 PMID: 31950903

16. Lancaster C, Teeters J, Gros D, Back S. Posttraumatic Stress Disorder: Overview of Evidence-Based Assessment and Treatment. J Clin Med [Internet]. 2016 Nov 22; 5(11):105. Available from: http://www.mdpi.com/2077-0383/5/11/105 PMID: 27879650

17. Xu KY, Goodman E, Goswami R, Crane M, Crowley L, Busse P, et al. Determinants of asthma morbidity in World Trade Center rescue and recovery workers. Ann Allergy, Asthma Immunol [Internet]. 2016 Nov; 117(5):568–70. Available from: https://linkinghub.elsevier.com/retrieve/pii/S108112061630624X https://doi.org/10.1016/j.anai.2016.08.033 PMID: 27788891

18. Chung MC, Rudd H, Wall N. Posttraumatic stress disorder following asthma attack (post-asthma attack PTSD) and psychiatric co-morbidity: The impact of alexithymia and coping. Psychiatry Res [Internet]. 2012 May; 197(3):246–52. Available from: https://linkinghub.elsevier.com/retrieve/pii/S0165178112000194 https://doi.org/10.1016/j.psychres.2012.01.008 PMID: 22424893

19. Wang T, Ji Y-L, Yang Y-Y, Xiong X-Y, Wang I-M, Sandford AJ, et al. Transcriptomic profiling of peripheral blood CD4+ T-cells in asthmatics with and without depression. Gene [Internet]. 2015 July; 565 (2):282–87. https://doi.org/10.1016/j.gene.2015.04.029 PMID: 25871513

20. Zhu Z, Zhu X, Liu C-L, Shi H, Shen S, Yang Y, et al. Shared genetics of asthma and mental health disorders: a large-scale genome-wide cross-trait analysis. Eur Respir J [Internet]. 2019 Dec; 54 (6):1901507. Available from: http://erj.ersjournals.com/lookup/doi/10.1183/13993003.01507-2019 PMID: 31619474

21. Cao H, Li S, Baranova A, Zhang F. Shared Genetic Liability Between Major Depressive Disorder and Atopic Diseases. Front Immunol [Internet]. 2021 Sep 8;12. Available from: https://www.frontiersin.org/articles/10.3389/fimmu.2021.665160/full PMID: 34566951

22. Nievergelt CM, Maihofer AX, Klengel T, Atkinson EG, Chen C-Y, Choi KW, et al. International meta-analysis of PTSD genome-wide association studies identifies sex- and ancestry-specific genetic risk loci. Nat Commun [Internet]. 2019 Dec 8; 10(1):4558. Available from: http://www.nature.com/articles/s41467-019-12576-w https://doi.org/10.1038/s41467-019-12576-w PMID: 31594949

23. Gautam Y., Johansson E., & Mersha T. B. Multi-Omics Profiling Approach to Asthma: An Evolving Paradigm. J. pers. Med [Internet]. 2022: 12(1), 66. Available from: https://doi.org/10.3390/jpm12010066 PMID: 35055381

24. Yan Q., Forno E., Cardenas A., Qi C., Han Y. Y., Acosta-Pérez E., et al. Exposure to violence, chronic stress, nasal DNA methylation, and atopic asthma in children. Pediatr. Pulmonol. [Internet]. 2021 Mar: 56(7), 1896–1905. Available from: https://doi.org/10.1002/ppul.25372 PMID: 33751861

25. Trump S., Bieg M., Gu Z., Thürmann L., Bauer T., Bauer M., et al. Prenatal maternal stress and wheeze in children: novel insights into epigenetic regulation. Sci. rep. 2016: 6, 28616. Available from: https://doi.org/10.1038/srep28616 PMID: 27349968

26. Fitzpatrick A. M., Park Y., Brown L. A., & Jones D. P. Children with severe asthma have unique oxidative stress-associated metabolomic profiles. J. Allergy Clin. Immunol. 2014: 133(1), 258–61.e618. Available from: https://doi.org/10.1016/j.jaci.2013.10.012 PMID: 24369802

27. Bravo J. A., Forsythe P., Chew M. V., Escaravage E., Savignac H. M., Dinan T. G., et al. (2011). Ingestion of Lactobacillus strain regulates emotional behavior and central GABA receptor expression in a mouse via the vagus nerve. *Proceedings of the National Academy of Sciences of the United States of America*, 108(38), 16050–16055. https://doi.org/10.1073/pnas.1102999108 PMID: 21876150

28. Bharwani A., Mian M. F., Surette M. G., Bienenstock J., & Forsythe P. (2017). Oral treatment with Lactobacillus rhamnosus attenuates behavioural deficits and immune changes in chronic social stress. BMC medicine, 15(1), 7. https://doi.org/10.1186/s12916-016-0771-7 PMID: 28073366

29. Liu Y., Mian M. F., McVey Neufeld K. A., & Forsythe P. (2020). $CD4^{+}CD25^{+}$ T Cells are Essential for Behavioral Effects of Lactobacillus rhamnosus JB-1 in Male BALB/c mice. *Brain, behavior, and immunity*, 88, 451–460. https://doi.org/10.1016/j.bbi.2020.04.014

30. Breslow DK, Collins SR, Bodenmiller B, Aebersold R, Simons K, Shevchenko A, et al. Orm family proteins mediate sphingolipid homeostasis. Nature [Internet]. 2010 Feb; 463(7284):1048–53. Available from: http://www.nature.com/articles/nature08787 PMID: 20182505

31. James B, Milstien S, Spiegel S. ORMDL3 and allergic asthma: From physiology to pathology. J Allergy Clin Immunol [Internet]. 2019 Sep; 144(3):634–40. Available from: https://linkinghub.elsevier.com/retrieve/pii/S0091674919309728 PMID: 31376405

32. Cai L, Oyeniran C, Biswas DD, Allegood J, Milstien S, Kordula T, et al. ORMDL proteins regulate ceramide levels during sterile inflammation. J Lipid Res [Internet]. 2016 Aug; 57(8):1412–22. Available from: https://linkinghub.elsevier.com/retrieve/pii/S0022227520352135 https://doi.org/10.1194/jlr.M065920 PMID: 27313060

33. Oyeniran C, Sturgill JL, Hait NC, Huang W-C, Avni D, Maceyka M, et al. Aberrant ORM (yeast)–like protein isoform 3 (ORMDL3) expression dysregulates ceramide homeostasis in cells and ceramide exacerbates allergic asthma in mice. J Allergy Clin Immunol [Internet]. 2015 Oct; 136(4):1035–1046.e6. Available from: https://linkinghub.elsevier.com/retrieve/pii/S0091674915003334 https://doi.org/10.1016/j.jaci.2015.02.031 PMID: 25842287

34. Ferreira MAR, McRae AF, Medland SE, Nyholt DR, Gordon SD, Wright MJ, et al. Association between ORMDL3, IL1RL1 and a deletion on chromosome 17q21 with asthma risk in Australia. Eur J Hum Genet [Internet]. 2011 Apr 8; 19(4):458–64. Available from: http://www.nature.com/articles/ejhg2010191 https://doi.org/10.1038/ejhg.2010.191 PMID: 21150878

35. Moffatt MF, Kabesch M, Liang L, Dixon AL, Strachan D, Heath S, et al. Genetic variants regulating ORMDL3 expression contribute to the risk of childhood asthma. Nature [Internet]. 2007 Jul 4; 448(7152):470–3. Available from: http://www.nature.com/articles/nature06014 https://doi.org/10.1038/nature06014 PMID: 17611496

36. Choudhry S, Taub M, Mei R, Rodriguez-Santana J, Rodriguez-Cintron W, Shriver MD, et al. Genome-wide screen for asthma in Puerto Ricans: evidence for association with 5q23 region. Hum Genet [Internet]. 2008 Jun 10; 123(5):455–68. Available from: http://link.springer.com/10.1007/s00439-008-0495-7 PMID: 18401594

37. Himes BE, Hunninghake GM, Baurley JW, Rafaels NM, Sleiman P, Strachan DP, et al. Genome-wide Association Analysis Identifies PDE4D as an Asthma-Susceptibility Gene. Am J Hum Genet [Internet]. 2009 May; 84(5):581–93. Available from: https://linkinghub.elsevier.com/retrieve/pii/S0002929709001487 https://doi.org/10.1016/j.ajhg.2009.04.006 PMID: 19426955

38. Hancock DB, Romieu I, Shi M, Sienra-Monge J-J, Wu H, Chiu GY, et al. Genome-Wide Association Study Implicates Chromosome 9q21.31 as a Susceptibility Locus for Asthma in Mexican Children. Gibson G, editor. PLoS Genet [Internet]. 2009 Aug 28; 5(8):e1000623. Available from: https://dx.plos.org/10.1371/journal.pgen.1000623 PMID: 19714205

39. Sleiman PMA, Flory J, Imielinski M, Bradfield JP, Annaiah K, Willis-Owen SAG, et al. Variants of DENND1B Associated with Asthma in Children. N Engl J Med [Internet]. 2010 Jan 7; 362(1):36–44. Available from: http://www.nejm.org/doi/abs/10.1056/NEJMoa0901867 PMID: 20032318

40. Mathias RA, Grant A V., Rafaels N, Hand T, Gao L, Vergara C, et al. A genome-wide association study on African-ancestry populations for asthma. J Allergy Clin Immunol [Internet]. 2010 Feb; 125(2):336–346.e4. Available from: https://linkinghub.elsevier.com/retrieve/pii/S0091674909013165 https://doi.org/10.1016/j.jaci.2009.08.031 PMID: 19910028

41. Li X, Howard TD, Zheng SL, Haselkorn T, Peters SP, Meyers DA, et al. Genome-wide association study of asthma identifies RAD50-IL13 and HLA-DR/DQ regions. J Allergy Clin Immunol [Internet]. 2010 Feb; 125(2):328–335.e11. Available from: https://linkinghub.elsevier.com/retrieve/pii/S0091674909017357 https://doi.org/10.1016/j.jaci.2009.11.018 PMID: 20159242

42. Masini E, Giannini L, Nistri S, Cinci L, Mastroianni R, Xu W, et al. Ceramide: a Key Signaling Molecule in a Guinea Pig Model of Allergic Asthmatic Response and Airway Inflammation. J Pharmacol Exp Ther [Internet]. 2008 Feb; 324(2):548–57. Available from: http://jpet.aspetjournals.org/lookup/doi/10.1124/jpet.107.131565 PMID: 18042827

43. Petrache I, Kamocki K, Poirier C, Pewzner-Jung Y, Laviad EL, Schweitzer KS, et al. Ceramide Synthases Expression and Role of Ceramide Synthase-2 in the Lung: Insight from Human Lung Cells and Mouse Models. Chu HW, editor. PLoS One [Internet]. 2013 May 14; 8(5):e62968. Available from: https://dx.plos.org/10.1371/journal.pone.0062968

44. Spassieva SD, Mullen TD, Townsend DM, Obeid LM. Disruption of ceramide synthesis by CerS2 down-regulation leads to autophagy and the unfolded protein response. Biochem J [Internet]. 2009 Dec 1; 424(2):273–83. Available from: https://portlandpress.com/biochemj/article/424/2/273/79924/Disruption-of-ceramide-synthesis-by-CerS2-down https://doi.org/10.1042/BJ20090699 PMID: 19728861

45. Epstein S, Kirkpatrick CL, Castillon GA, Muñiz M, Riezman I, David FPA, et al. Activation of the unfolded protein response pathway causes ceramide accumulation in yeast and INS-1E insulinoma cells. J Lipid Res [Internet]. 2012 Mar; 53(3):412–20. Available from: https://linkinghub.elsevier.com/retrieve/pii/S0022227520413562 https://doi.org/10.1194/jlr.M022186 PMID: 22210926

46. Choi S, Snider JM, Olakkengil N, Lambert JM, Anderson AK, Ross-Evans JS, et al. Myristate-induced endoplasmic reticulum stress requires ceramide synthases 5/6 and generation of C14-ceramide in intestinal epithelial cells. FASEB J [Internet]. 2018 Oct 16; 32(10):5724–36. Available from: https://onlinelibrary.wiley.com/doi/abs/10.1096/fj.201800141R PMID: 29768040

47. Ha SG, Ge XN, Bahaie NS, Kang BN, Rao A, Rao SP, et al. ORMDL3 promotes eosinophil trafficking and activation via regulation of integrins and CD48. Nat Commun [Internet]. 2013 Dec 23; 4(1):2479. Available from: http://www.nature.com/articles/ncomms3479 https://doi.org/10.1038/ncomms3479 PMID: 24056518

48. Miller M, Tam AB, Mueller JL, Rosenthal P, Beppu A, Gordillo R, et al. Cutting Edge: Targeting Epithelial ORMDL3 Increases, Rather than Reduces, Airway Responsiveness and Is Associated with Increased Sphingosine-1-Phosphate. J Immunol [Internet]. 2017 Apr 15; 198(8):3017–22. Available from: http://www.jimmunol.org/lookup/doi/10.4049/jimmunol.1601848 PMID: 28275141

49. Bugajev V, Halova I, Draberova L, Bambouskova M, Potuckova L, Draberova H, et al. Negative regulatory roles of ORMDL3 in the FcεRI-triggered expression of proinflammatory mediators and chemotactic response in murine mast cells. Cell Mol Life Sci [Internet]. 2016 Mar 25; 73(6):1265–85. Available from: http://link.springer.com/10.1007/s00018-015-2047-3 PMID: 26407610

50. Mencarelli C, Martinez–Martinez P. Ceramide function in the brain: when a slight tilt is enough. Cell Mol Life Sci [Internet]. 2013 Jan 24; 70(2):181–203. Available from: http://link.springer.com/10.1007/s00018-012-1038-x PMID: 22729185

51. Kornhuber J, Gulbins E. New Molecular Targets for Antidepressant Drugs. Pharmaceuticals [Internet]. 2021 Sep 2; 14(9):894. Available from: https://www.mdpi.com/1424-8247/14/9/894 https://doi.org/10.3390/ph14090894 PMID: 34577594

52. Gulbins E, Palmada M, Reichel M, Lüth A, Böhmer C, Amato D, et al. Acid sphingomyelinase–ceramide system mediates effects of antidepressant drugs. Nat Med [Internet]. 2013 Jul 16; 19(7):934–8. Available from: http://www.nature.com/articles/nm.3214 https://doi.org/10.1038/nm.3214 PMID: 23770692

53. Chen B, Zhao L, Li X, Ji Y-S, Li N, Xu X-F, et al. Syntaxin 8 Modulates the Post-synthetic Trafficking of the TrkA Receptor and Inflammatory Pain Transmission*. J Biol Chem [Internet]. 2014 Jul; 289 (28):19556–69. Available from: https://linkinghub.elsevier.com/retrieve/pii/S0021925820477208 https://doi.org/10.1074/jbc.M114.567925 PMID: 24872407

54. Piedimonte G. Contribution of neuroimmune mechanisms to airway inflammation and remodeling during and after respiratory syncytial virus infection. Pediatr Infect Dis J [Internet]. 2003 Feb; 22(Supplement):S66–75. Available from: http://journals.lww.com/00006454-200302001-00010

55. Prakash Y, Thompson MA, Meuchel L, Pabelick CM, Mantilla CB, Zaidi S, et al. Neurotrophins in lung health and disease. Expert Rev Respir Med [Internet]. 2010 Jun 9; 4(3):395–411. Available from: http://www.tandfonline.com/doi/full/10.1586/ers.10.29 PMID: 20524922

56. Prakash YS, Thompson MA, Pabelick CM. Brain-Derived Neurotrophic Factor in TNF-α Modulation of Ca 2+ in Human Airway Smooth Muscle. Am J Respir Cell Mol Biol [Internet]. 2009 Nov; 41(5):603–11. Available from: http://www.atsjournals.org/doi/abs/10.1165/rcmb.2008-0151OC

57. Micera A, Vigneti E, Pickholtz D, Reich R, Pappo O, Bonini S, et al. Nerve growth factor displays stimulatory effects on human skin and lung fibroblasts, demonstrating a direct role for this factor in tissue repair. Proc Natl Acad Sci [Internet]. 2001 May 22; 98(11):6162–7. Available from: https://pnas.org/doi/full/10.1073/pnas.101130898

58. Feng D-Y, Guo B-L, Liu G-H, Xu K, Yang J, Tao K, et al. Nerve growth factor against PTSD symptoms: Preventing the impaired hippocampal cytoarchitectures. Prog Neurobiol [Internet]. 2020 Jan; 184:101721. Available from: https://linkinghub.elsevier.com/retrieve/pii/S0301008219303399 https://doi.org/10.1016/j.pneurobio.2019.101721 PMID: 31704315

59. Bilan F, Thoreau V, Nacfer M, Dérand R, Norez C, Cantereau A, et al. Syntaxin 8 impairs trafficking of cystic fibrosis transmembrane conductance regulator (CFTR) and inhibits its channel activity. J Cell Sci [Internet]. 2004 Apr 15; 117(10):1923–35. Available from: https://journals.biologists.com/jcs/article/117/10/1923/27738/Syntaxin-8-impairs-trafficking-of-cystic-fibrosis

60. Dahl M, Tybærg-Hansen A, Lange P, Nordestgaard BG. ΔF508 heterozygosity in cystic fibrosis and susceptibility to asthma. Lancet [Internet]. 1998 Jun; 351(9120):1911–3. Available from: https://linkinghub.elsevier.com/retrieve/pii/S0140673697114192

61. Dahl M, Nordestgaard BG, Lange P, Tybjaerg-Hansen A. Fifteen-year follow-up of pulmonary function in individuals heterozygous for the cystic fibrosis phenylalanine-508 deletion. J Allergy Clin Immunol [Internet]. 2001 May; 107(5):818–23. Available from: https://linkinghub.elsevier.com/retrieve/pii/S0091674901141187 https://doi.org/10.1067/mai.2001.114117 PMID: 11344348

62. Douros K, Loukou I, Doudounakis S, Tzetis M, Priftis KN, Kanavakis E. Asthma and pulmonary function abnormalities in heterozygotes for cystic fibrosis transmembrane regulator gene mutations. Int J

Clin Exp Med [Internet]. 2008; 1(4):345–9. Available from: http://www.ncbi.nlm.nih.gov/pubmed/19079680 PMID: 19079680

63. Reznikov LR. Cystic Fibrosis and the Nervous System. Chest [Internet]. 2017 May; 151(5):1147–55. Available from: https://linkinghub.elsevier.com/retrieve/pii/S0012369216623565 https://doi.org/10.1016/j.chest.2016.11.009 PMID: 27876591

64. Lavelin I, Geiger B. Characterization of a Novel GTPase-activating Protein Associated with Focal Adhesions and the Actin Cytoskeleton. J Biol Chem [Internet]. 2005 Feb; 280(8):7178–85. Available from: https://linkinghub.elsevier.com/retrieve/pii/S002192581962836X PMID: 15611138

65. Chiba Y, Ueno A, Shinozaki K, Takeyama H, Nakazawa S, Sakai H, et al. Involvement of RhoA-mediated Ca2+ sensitization in antigen-induced bronchial smooth muscle hyperresponsiveness in mice. Respir Res [Internet]. 2005 Dec 8; 6(1):4. Available from: http://respiratory-research.biomedcentral.com/articles/ https://doi.org/10.1186/1465-9921-6-4 PMID: 15638941

66. Diana G, Valentini G, Travaglione S, Falzano L, Pieri M, Zona C, et al. Enhancement of learning and memory after activation of cerebral Rho GTPases. Proc Natl Acad Sci [Internet]. 2007 Jan 9; 104 (2):636–41. Available from: https://pnas.org/doi/full/10.1073/pnas.0610059104

67. den Hollander P, Rawls K, Tsimelzon A, Shepherd J, Mazumdar A, Hill J, et al. Phosphatase PTP4A3 Promotes Triple-Negative Breast Cancer Growth and Predicts Poor Patient Survival. Cancer Res [Internet]. 2016 Apr 1; 76(7):1942–53. Available from: http://cancerres.aacrjournals.org/lookup/doi/10.1158/0008-5472.CAN-14-0673 PMID: 26921331

68. Pei J, Grishin N V. Unexpected diversity in Shisa-like proteins suggests the importance of their roles as transmembrane adaptors. Cell Signal [Internet]. 2012 Mar 1; 24(3):758–69. Available from: http://www.ncbi.nlm.nih.gov/pubmed/26921331 https://doi.org/10.1016/j.cellsig.2011.11.011 PMID: 22120523

69. Oláh J, Lehotzky A, Szunyogh S, Szénási T, Orosz F, Ovádi J. Microtubule-Associated Proteins with Regulatory Functions by Day and Pathological Potency at Night. Cells [Internet]. 2020 Feb 4; 9 (2):357. Available from: https://www.mdpi.com/2073-4409/9/2/357

70. Bigler J, Boedigheimer M, Schofield JPR, Skipp PJ, Corfield J, Rowe A, et al. A Severe Asthma Disease Signature from Gene Expression Profiling of Peripheral Blood from U-BIOPRED Cohorts. Am J Respir Crit Care Med [Internet]. 2017 May 15; 195(10):1311–20. Available from: http://www.atsjournals.org/doi/10.1164/rccm.201604-0866OC PMID: 27925796

71. Rusch HL, Robinson J, Yun S, Osier ND, Martin C, Brewin CR, et al. Gene expression differences in PTSD are uniquely related to the intrusion symptom cluster: A transcriptome-wide analysis in military service members. Brain Behav Immun [Internet]. 2019 Aug; 80:904–8. Available from: https://linkinghub.elsevier.com/retrieve/pii/S0889159119301540 https://doi.org/10.1016/j.bbi.2019.04.039 PMID: 31039430

72. Kuan P-F, Waszczuk MA, Kotov R, Clouston S, Yang X, Singh PK, et al. Gene expression associated with PTSD in World Trade Center responders: An RNA sequencing study. Transl Psychiatry [Internet]. 2017 Dec 18; 7(12):1297. Available from: http://www.nature.com/articles/s41398-017-0050-1 https://doi.org/10.1038/s41398-017-0050-1 PMID: 29249826

73. Ioannidis I, McNally B, Willette M, Peeples ME, Chaussabel D, Durbin JE, et al. Plasticity and Virus Specificity of the Airway Epithelial Cell Immune Response during Respiratory Virus Infection. J Virol [Internet]. 2012 May 15; 86(10):5422–36. Available from: https://journals.asm.org/doi/10.1128/JVI.06757-11 PMID: 22398282

74. Franscina Pinto E, Andrade C. Interferon-Related Depression: A Primer on Mechanisms, Treatment, and Prevention of a Common Clinical Problem. Curr Neuropharmacol [Internet]. 2016 Aug 26; 14 (7):743–8. Available from: http://www.eurekaselect.com/openurl/content.php?genre=article&issn=1570-159X&volume=14&issue=7&spage=743

75. Zheng L-S, Hitoshi S, Kaneko N, Takao K, Miyakawa T, Tanaka Y, et al. Mechanisms for Interferon-α-Induced Depression and Neural Stem Cell Dysfunction. Stem Cell Reports [Internet]. 2014 Jul; 3 (1):73–84. Available from: https://linkinghub.elsevier.com/retrieve/pii/S2213671114001568 https://doi.org/10.1016/j.stemcr.2014.05.015 PMID: 25068123

76. Rich HE, Antos D, Melton NR, Alcorn JF, Manni ML. Insights Into Type I and III Interferons in Asthma and Exacerbations. Front Immunol [Internet]. 2020; 11:574027. Available from: http://www.ncbi.nlm.nih.gov/pubmed/33101299 https://doi.org/10.3389/fimmu.2020.574027 PMID: 33101299

77. Gomez JL, Diaz MP, Nino G, Britto CJ. Impaired type I interferon regulation in the blood transcriptome of recurrent asthma exacerbations. BMC Med Genomics [Internet]. 2018 Dec 27; 11(1):21. Available from: https://bmcmedgenomics.biomedcentral.com/articles/10.1186/s12920-018-0340-3 PMID: 29486764

78. Abbas AR, Baldwin D, Ma Y, Ouyang W, Gurney A, Martin F, et al. Immune response in silico (IRIS): immune-specific genes identified from a compendium of microarray expression data. Genes Immun

[Internet]. 2005 Jun 1; 6(4):319–31. Available from: https://www.nature.com/articles/6364173 https://doi.org/10.1038/sj.gene.6364173 PMID: 15789058

79. Atli A, Demir S, Bulut M, Okan İbiloğlu A, Güneş M, Kaya M, et al. Neutrophil–lymphocyte ratio in patients with major depressive disorder undergoing no pharmacological therapy. Neuropsychiatr Dis Treat [Internet]. 2015 Aug;2253. Available from: http://www.dovepress.com/neutrophilndashlymphocyte-ratio-in-patients-with-major-depressive-diso-peer-reviewed-article-NDT

80. Gao H, Ying S, Dai Y. Pathological Roles of Neutrophil-Mediated Inflammation in Asthma and Its Potential for Therapy as a Target. J Immunol Res [Internet]. 2017; 2017:1–12. Available from: https://www.hindawi.com/journals/jir/2017/3743048/ https://doi.org/10.1155/2017/3743048 PMID: 29359169

81. Ahmetspahic D, Schwarte K, Ambrée O, Bürger C, Falcone V, Seiler K, et al. Altered B Cell Homeostasis in Patients with Major Depressive Disorder and Normalization of CD5 Surface Expression on Regulatory B Cells in Treatment Responders. J Neuroimmune Pharmacol [Internet]. 2018 Mar 13; 13 (1):90–9. Available from: http://link.springer.com/10.1007/s11481-017-9763-4 PMID: 28905187

82. Habener A, Happle C, Grychtol R, Skuljec J, Busse M, Dalüge K, et al. Regulatory B cells control airway hyperreactivity and lung remodeling in a murine asthma model. J Allergy Clin Immunol [Internet]. 2021 Jun; 147(6):2281–2294.e7. Available from: https://linkinghub.elsevier.com/retrieve/pii/S0091674920316341 https://doi.org/10.1016/j.jaci.2020.09.041 PMID: 33249168

83. Ghosh S, Hoselton SA, Asbach S V, Steffan BN, Wanjara SB, Dorsam GP, et al. B lymphocytes regulate airway granulocytic inflammation and cytokine production in a murine model of fungal allergic asthma. Cell Mol Immunol [Internet]. 2015 Mar 3; 12(2):202–12. Available from: http://www.nature.com/articles/cmi2014103 https://doi.org/10.1038/cmi.2014.103 PMID: 25363529

84. De Vooght V, Carlier V, Devos FC, Haenen S, Verbeken E, Nemery B, et al. B-lymphocytes as Key Players in Chemical-Induced Asthma. Shiku H, editor. PLoS One [Internet]. 2013 Dec 13; 8(12): e83228. Available from: https://dx.plos.org/10.1371/journal.pone.0083228 PMID: 24349469

85. Alvarez-Mon MA, Gómez AM, Orozco A, Lahera G, Sosa MD, Diaz D, et al. Abnormal Distribution and Function of Circulating Monocytes and Enhanced Bacterial Translocation in Major Depressive Disorder. Front Psychiatry [Internet]. 2019 Nov 15; 10. Available from: https://www.frontiersin.org/article/10.3389/fpsyt.2019.00812/full PMID: 31803077

86. Seidel A, Arolt V, Hunstiger M, Rink L, Behnisch A, Kirchner H. Major depressive disorder is associated with elevated monocyte counts. Acta Psychiatr Scand [Internet]. 1996 Sep; 94(3):198–204. Available from: https://onlinelibrary.wiley.com/doi/10.1111/j.1600-0447.1996.tb09849.x PMID: 8891088

87. Niessen NM, Baines KJ, Simpson JL, Scott HA, Qin L, Gibson PG, et al. Neutrophilic asthma features increased airway classical monocytes. Clin Exp Allergy [Internet]. 2021 Feb; 51(2):305–17. Available from: https://onlinelibrary.wiley.com/doi/10.1111/cea.13811 PMID: 33301598

88. Tanizaki Y, Hosokawa M, Goda Y, Akagi K, Takeyama H, Kimura I. Numerical changes in blood monocytes in bronchial asthma. Acta Med Okayama [Internet]. 1982 Oct; 36(5):341–8. Available from: http://www.ncbi.nlm.nih.gov/pubmed/7180570 https://doi.org/10.18926/AMO/30688 PMID: 7180570

89. DeBaun MR, Strunk RC. The intersection between asthma and acute chest syndrome in children with sickle-cell anaemia. Lancet [Internet]. 2016 Jun; 387(10037):2545–53. Available from: https://linkinghub.elsevier.com/retrieve/pii/S0140673616001458 https://doi.org/10.1016/S0140-6736(16)00145-8 PMID: 27353685

90. Jison ML, Munson PJ, Barb JJ, Suffredini AF, Talwar S, Logun C, et al. Blood mononuclear cell gene expression profiles characterize the oxidant, hemolytic, and inflammatory stress of sickle cell disease. Blood [Internet]. 2004 Jul 1; 104(1):270–80. Available from: https://ashpublications.org/blood/article/104/1/270/114489/Blood-mononuclear-cell-gene-expression-profiles https://doi.org/10.1182/blood-2003-08-2760 PMID: 15031206

91. Liu S., Huang Z., Deng X., Zou X., Li H., Mu S., et al. (2021). Identification of key candidate biomarkers for severe influenza infection by integrated bioinformatical analysis and initial clinical validation. *Journal of cellular and molecular medicine*, 25(3), 1725–1738. https://doi.org/10.1111/jcmm.16275 PMID: 33448094

92. Yang Z., Ni J., Kuang L., Gao Y., & Tao S. (2020). Identification of genes and pathways associated with subchondral bone in osteoarthritis via bioinformatic analysis. *Medicine*, 99(37), e22142. https://doi.org/10.1097/MD.0000000000022142 PMID: 32925767

93. Sinha P., & Calfee C. S. (2019). Peeking under the Hood of Acute Respiratory Distress Syndrome Phenotypes: Deeper Insights into Biological Heterogeneity. *American journal of respiratory and critical care medicine*, 200(1), 4–6. https://doi.org/10.1164/rccm.201901-0195ED PMID: 30753791

94. Gong F., Li R., Zheng X., Chen W., Zheng Y., Yang Z., et al. (2021). OLFM4 Regulates Lung Epithelial Cell Function in Sepsis-Associated ARDS/ALI via LDHA-Mediated NF-κB Signaling. *Journal of inflammation research*, 14, 7035–7051. https://doi.org/10.2147/JIR.S335915 PMID: 34955649

95. Zhang N., Aiyasiding X., Li W. J., Liao H. H., & Tang Q. Z. (2022). Neutrophil degranulation and myocardial infarction. *Cell communication and signaling: CCS*, 20(1), 50. https://doi.org/10.1186/s12964-022-00824-4 PMID: 35410418

96. Robledo S., Idol R. A., Crimmins D. L., Ladenson J. H., Mason P. J., & Bessler M. (2008). The role of human ribosomal proteins in the maturation of rRNA and ribosome production. RNA (New York, N.Y.), 14(9), 1918–1929. https://doi.org/10.1261/rna.1132008 PMID: 18697920

97. Gan S. J., Ye B., Qian S. X., Zhang C., Mao J. Q., Li K., et al. (2015). Immune- and ribosome-related genes were associated with systemic vasculitis. *Scandinavian journal of immunology*, 81(2), 96–101. https://doi.org/10.1111/sji.12252 PMID: 25410188

98. Quarello P., Garelli E., Carando A., Brusco A., Calabrese R., Dufour C., et al. (2010). Diamond-Blackfan anemia: genotype-phenotype correlations in Italian patients with RPL5 and RPL11 mutations. *Haematologica*, 95(2), 206–213. https://doi.org/10.3324/haematol.2009.011783 PMID: 19773262

99. Konno Y., Toki T., Tandai S., Xu G., Wang R., Terui K., et al. (2010). Mutations in the ribosomal protein genes in Japanese patients with Diamond-Blackfan anemia. *Haematologica*, 95(8), 1293–1299. https://doi.org/10.3324/haematol.2009.020826 PMID: 20378560

100. Ahamad J., Ojha S., Srivastava A., Bhattacharya A., & Bhattacharya S. (2015). Post-transcriptional regulation of ribosomal protein genes during serum starvation in Entamoeba histolytica. *Molecular and biochemical parasitology*, 201(2), 146–152. https://doi.org/10.1016/j.molbiopara.2015.07.006 PMID: 26247142

101. Kewalramani A., Bollinger M. E., & Postolache T. T. (2008). Asthma and Mood Disorders. *International journal of child health and human development: IJCHD*, 1(2), 115–123. PMID: 19180246

102. O'Toole BI, Catts S V. Trauma, PTSD, and physical health: An epidemiological study of Australian Vietnam veterans. J Psychosom Res [Internet]. 2008 Jan; 64(1):33–40. Available from: https://linkinghub.elsevier.com/retrieve/pii/S0022399907002899

103. Allgire E, McAlees JW, Lewkowich IP, Sah R. Asthma and posttraumatic stress disorder (PTSD): Emerging links, potential models and mechanisms. Brain Behav Immun [Internet]. 2021 Oct; 97:275–85. Available from: https://linkinghub.elsevier.com/retrieve/pii/S0889159121002324 https://doi.org/10.1016/j.bbi.2021.06.001 PMID: 34107349

104. Leday GGR, Vértes PE, Richardson S, Greene JR, Regan T, Khan S, et al. Replicable and Coupled Changes in Innate and Adaptive Immune Gene Expression in Two Case-Control Studies of Blood Microarrays in Major Depressive Disorder. Biol Psychiatry [Internet]. 2018 Jan; 83(1):70–80. Available from: https://linkinghub.elsevier.com/retrieve/pii/S0006322317316177 https://doi.org/10.1016/j.biopsych.2017.01.021 PMID: 28688579

105. Ritchie ME, Phipson B, Wu D, Hu Y, Law CW, Shi W, et al. limma powers differential expression analyses for RNA-sequencing and microarray studies. Nucleic Acids Res [Internet]. 2015 Apr 20; 43(7): e47–e47. Available from: http://academic.oup.com/nar/article/43/7/e47/2414268/limma-powers-differential-expression-analyses-for https://doi.org/10.1093/nar/gkv007 PMID: 25605792

106. Spijker S, Van Zanten JS, De Jong S, Penninx BWJH, van Dyck R, Zitman FG, et al. Stimulated Gene Expression Profiles as a Blood Marker of Major Depressive Disorder. Biol Psychiatry [Internet]. 2010 Jul; 68(2):179–86. Available from: https://linkinghub.elsevier.com/retrieve/pii/S0006322310002465 https://doi.org/10.1016/j.biopsych.2010.03.017 PMID: 20471630

107. Wilhelm BT, Marguerat S, Watt S, Schubert F, Wood V, Goodhead I, et al. Dynamic repertoire of a eukaryotic transcriptome surveyed at single-nucleotide resolution. Nature [Internet]. 2008 Jun 18; 453 (7199):1239–43. Available from: http://www.nature.com/articles/nature07002 https://doi.org/10.1038/nature07002 PMID: 18488015

108. Xu X, Zhang Y, Williams J, Antoniou E, McCombie WR, Wu S, et al. Parallel comparison of Illumina RNA-Seq and Affymetrix microarray platforms on transcriptomic profiles generated from 5-aza-deoxycytidine treated HT-29 colon cancer cells and simulated datasets. BMC Bioinformatics [Internet]. 2013 Jun 28; 14(S9):S1. Available from: https://bmcbioinformatics.biomedcentral.com/articles/10.1186/1471-2105-14-S9-S1 PMID: 23902433

109. Robinson MD, McCarthy DJ, Smyth GK. edgeR: a Bioconductor package for differential expression analysis of digital gene expression data. Bioinformatics [Internet]. 2010 Jan 1; 26(1):139–40. Available from: https://academic.oup.com/bioinformatics/article-lookup/doi/10.1093/bioinformatics/btp616 PMID: 19910308

110. Wickham H. ggplot2 [Internet]. New York, NY: Springer New York; 2009. Available from: http://link.springer.com/10.1007/978-0-387-98141-3

111. Chen H, Boutros PC. VennDiagram: a package for the generation of highly-customizable Venn and Euler diagrams in R. BMC Bioinformatics [Internet]. 2011 Dec 26; 12(1):35. Available from: https://bmcbioinformatics.biomedcentral.com/articles/10.1186/1471-2105-12-35 PMID: 21269502

112. Luo W, Friedman MS, Shedden K, Hankenson KD, Woolf PJ. GAGE: generally applicable gene set enrichment for pathway analysis. BMC Bioinformatics [Internet]. 2009 Dec 27; 10(1):161. Available from: https://bmcbioinformatics.biomedcentral.com/articles/10.1186/1471-2105-10-161 PMID: 19473525

113. Blighe K, Rana S, Lewis M. EnhancedVolcano: Publication-ready volcano plots with enhanced colouring and labeling [Internet]. 2018. Available from: https://github.com/kevinblighe/EnhancedVolcano.
